# Participant experiences using novel home-based blood collection device for viral load testing in the HIV cure trials with analytical treatment interruptions

**Published:** 2022-08-02

**Authors:** Karine Dubé, Harsh Agarwal, William B. Carter, Lynda Dee, Jeff Taylor, Christopher Roebuck, Beth Peterson, Hursch Patel, Samuel Ndukwe, Kenneth M. Lynn, Linden Lalley-Chareczko, Emily Hiserodt, Sukyung Kim, Daniel Rosenbloom, Brad R. Evans, Melanie Anderson, Daria J. Hazuda, Kevin Bateman, Bonnie J. Howell, Livio Azzoni, Karam Mounzer, Pablo Tebas, Luis J. Montaner

**Affiliations:** 1 UNC Gillings School of Global Public Health, Chapel Hill, NC, USA; 2 BEAT-HIV Delaney Collaboratory Community Advisory Board (CAB), Philadelphia, PA, USA; 3 AIDS Treatment Activists Coalition (ATAC), Nationwide, Baltimore, MD, USA; 4 AIDS Action Baltimore, Baltimore, MD, USA; 5 Delaney AIDS Research Enterprise (DARE) CAB, San Francisco, CA, USA; 6 RID-HIV Collaboratory CAB, San Diego, CA, USA; 7 HIV + Aging Research Project – Palm Springs (HARP-PS), Palm Springs, CA, USA; 8 Department of Science and Technology Studies, Cornell University, Ithaca, NY, USA; 9 Wistar Institute and BEAT-HIV Delaney Collaboratory, Philadelphia, PA, USA; 10 Hospital of the University of Pennsylvania, Philadelphia, PA, USA; 11 Philadelphia FIGHT Community Health Centers, Philadelphia, PA, USA; 12 Merck & Co, Inc., Kenilworth, NJ, USA

**Keywords:** Home-based blood collection, experimental device, self-test, viral load, participant experiences, acceptability, HIV cure research, analytical treatment interruptions, people with HIV

## Abstract

**Background:**

HIV cure-directed clinical trials using analytical treatment interruptions (ATIs) require participants to adhere to frequent monitoring visits for viral load tests. Novel viral load monitoring strategies are needed to decrease participant burden during ATIs.

**Objective:**

To examine acceptability of a novel home-based blood collection device for viral load testing in the context of two ongoing ATI trials in Philadelphia, PA, United States.

**Methods:**

From January 2021 to February 2022, participants completed three in-depth interviews via teleconference during their participation in an ATI: (1) within two weeks of enrollment in the device study, (2) approximately four weeks after beginning to use the device, and (3) within two weeks of the end of the ATI when ART was re-initiated. We used conventional content analysis to analyze the data.

**Results:**

We recruited 17 participants: 15 were cisgender males, 1 cisgender female, and 1 transgender woman. We observed an overall 87% success rate in drawing blood with the device from home collection and found overall high acceptance of the device. A mean of 91.5 devices per participant were used for home-based blood collection. Most PWH viewed the device as relatively convenient, painless, easy to use, and a simple solution to frequent blood draws. The main challenge encountered was the inability to completely fill up devices with blood in some cases. Most participants reported positive experiences with mailing blood samples and could see themselves using the device on a regular basis outside of ATIs.

**Conclusions:**

Our study showed participant valued the novel home-based peripheral blood collection for viral load testing in the context of ATI trials. More research will be necessary to optimize implementation of the device and to assess whether blood collected can reliably measure viral loads in the context of ATI trials.

## Introduction

The field of HIV cure-directed research currently relies upon analytical treatment interruptions (ATIs) to determine the efficacy of interventions aimed at keeping HIV suppressed in the absence of antiretroviral treatment (ART).^1^ During ATIs, HIV is expected to return due to rebound competency. There is currently no known biomarker available that can accurately predict the likely time of the virus rebounding.^2-4^ ATI trials require study participants to adhere to frequent clinical monitoring visits for viral load tests which can be burdensome. These time commitments in turn may limit the number of people with HIV (PWH) who are willing to participate in trials. Further, repeated venipunctures during ATIs may carry risks such as pain and bruising. Novel viral load monitoring strategies are needed to decrease participant burden during ATI trials.^5,6^

**Figure 1:**
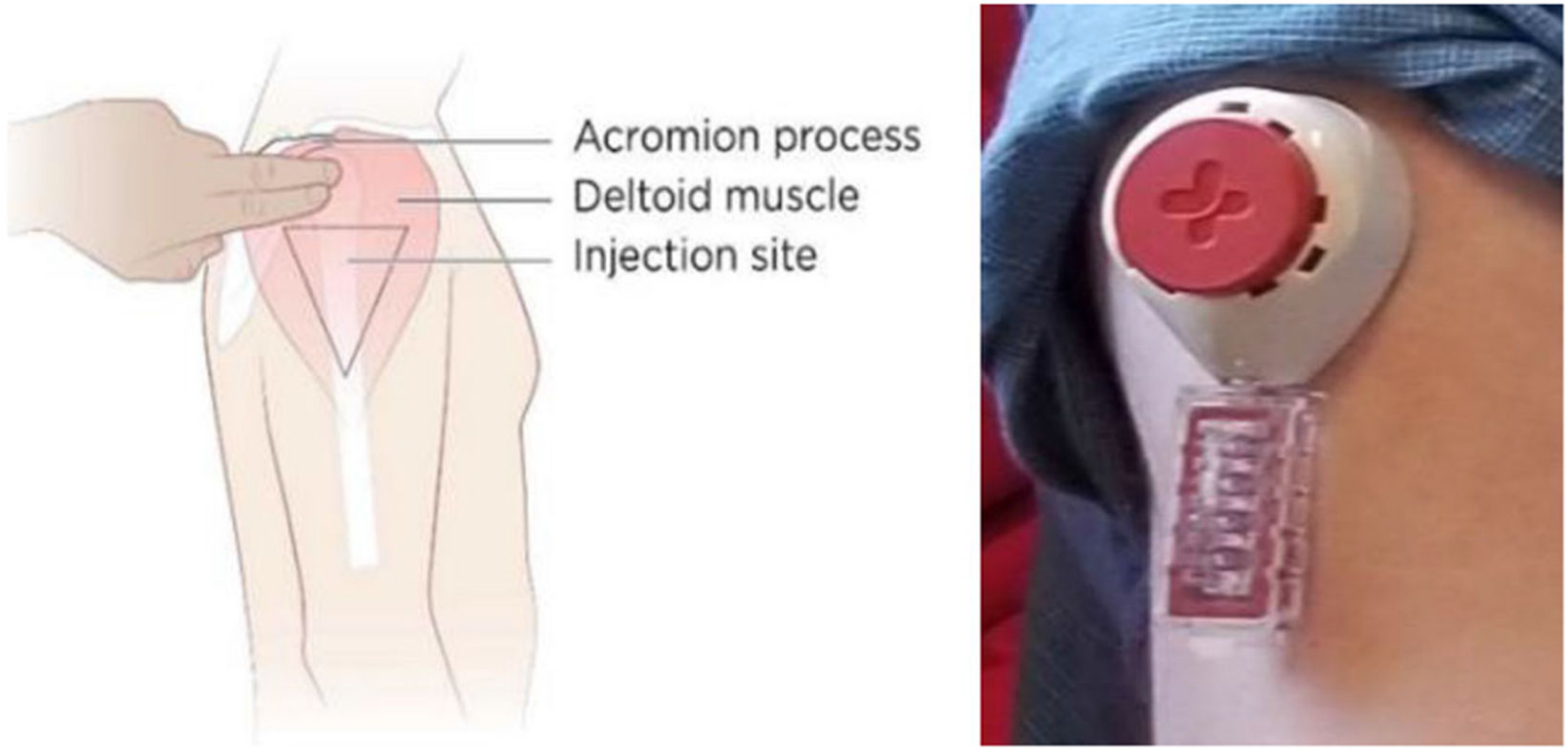
Experimental blood collection device for home-based viral load testing under development by Tasso, Inc. and Merck & Co, Inc., Kenilworth, NJ, USA.

Tasso, Inc.^7^ and Merck & Co, Inc. Kenilworth, NJ, USA developed a home-based blood collection device that could facilitate on-demand viral load testing during ATI trials. The Tasso device could avoid the need for venipuncture or open finger puncture for blood collection and thereby make ATIs more acceptable to PWH.^8^ The device is hand-held, creates a tight juncture to the skin and uses capillary action to enable self-collection of around 80 microliters of whole liquid blood in approximately five minutes (Figure 1).

Patient-centric remote specimen collections have also been explored for tuberculosis,^9-11^ hypertension and cardiovascular health,^12,13^ and have been available for diabetes since the 1970s.^14^ During the COVID-19 pandemic, home-based testing mechanisms gained popularity, becoming attractive options for sexually transmitted infection (STI) diagnosis.^15-24^ Specific to the field of HIV, antibody self-testing has been available for over two decades.^25-31^ There has been increased acceptability of home-based pre-exposure prophylaxis (PrEP) initiation, monitoring and support.^32^

The ability to test viral load from home may promote the acceptability of clinical trials involving ATIs. In a 2019 published international survey on attitudes towards ATIs, 59% of PWH (N = 442) expressed willingness to interrupt ART if home-based viral load testing was available.^33^ There is also growing consensus that such a home-based device could become a critical adjunct for PWH undergoing ATIs.^1^ Our socio-behavioral sciences research has shown the hypothetical acceptability of home-based blood collection for viral load testing in the context of ATIs.^34,35^ To date, however, no study has provided an in-depth examination of PWH’s experiences with home-based blood collection in the context of ATI trials.

We implemented an in-depth interview study to examine the acceptability of the Tasso^7^ home-based blood collection device when used during ongoing HIV cure-directed clinical trials including an ATI. Our qualitative study was nested as part of two ATI trials conducted by the BEAT-HIV Delaney Collaboratory Towards an HIV-1 cure (beat-hiv.org) based in Philadelphia, PA, USA. Our study objectives were to gain insight into (1) PWH’s overall perceptions and experiences with the device; (2) PWH’s recommendations to improve the device, (3) PWH’s experiences with mailing in samples, and (4) additional considerations to improve participant experiences. Findings can inform future refinement and implementation of the device in the context of ATI trials.

## Materials and methods

### Study setting and participant recruitment

Participants were referred to join the device acceptability study as part of two HIV cure-directed clinical trials involving an ATI conducted by the BEAT-HIV Martin Delaney Collaboratory (beat-hiv.org). The first clinical trial was titled *A Phase I Study to Evaluate Innate Activation Predictors of Sustained Viral Control in Adults Living with HIV Undergoing a Brief Analytical Treatment Interruption after the Administration of Pegylated Interferon Alpha 2b in Combination with Two Intravenous Broadly HIV-1 Neutralizing Antibodies 3BNC117 and 10-1074* (NCT03588715) and was conducted at Penn Presbyterian Medical Center and Philadelphia FIGHT Community Health Centers. The second clinical trial was titled *CD4 CAR + Zinc Finger Nuclease (ZFN)-modified T Cells in HIV Therapy* (NCT03617198) and was conducted at the Hospital of the University of Pennsylvania. To join the device acceptability component, participants had to be ≥ 18 years old, willing to provide informed consent and share their opinions about the blood collection experience.

### Data collection

We used qualitative research to explore perceptions of and experiences with the home-based blood collection device for viral load testing.^36^ We designed in-depth interview guides (Supplementary Appendix 1) in close collaboration with the BEAT-HIV Community Advisory Board (CAB) and Social Sciences Working Group. In addition, two community members from the AIDS Treatment Activists Coalition (ATAC) who were also members of the Delaney AIDS Research Enterprise (DARE) CAB provided input into the interview guides. We asked questions about participants’ decision to join the device study (initial interview only), reactions about the device, experiences with testing the device (e.g. perceived ease of use, comfort, safety, and pain), factors that would promote use versus barriers to using the device, and suggestions to improve the device and the device study. In addition, we elicited feedback on experiences with mailing samples, communication of viral load test results, perceived importance of being able to test viral load from home, possible effects on HIV stigma and social risks, and additional potential uses of the device besides viral load testing.

Interviews took place over a period of 14 months, from January 2021 to February 2022. We conducted in-depth interviews at three different timepoints: (1) within two weeks of enrollment in the device study; (2) approximately four (4) weeks after starting to use the device; and (3) within two weeks of the end of the ATI period when ART is re-initiated. Of note, two participants (TASSO-8 and TASSO-12) were post-intervention controllers and completed their interview at study closure while still on a monitored ATI. We conducted interviews in English, using a Health Insurance Portability and Accountability Act (HIPAA)-compliant conferencing platform. Interviews took between 20 and 60 minutes to complete. Participants received $50 for each interview completed.

Prior to using the device, all participants received orientation at the clinic about the device. In addition, they were instructed to watch the device demonstration video (https://vimeo.com/403592580). The video provided a description of the device and kit contents. Each device contained: instructions for use, the Tasso M-50 device, an alcohol wipe, a band-aid, a return pouch and a box. Participants were asked to use the device during their regular clinical study visits to compare viral load test results from the device versus viral loads obtained at the clinic. In addition, they were asked to use the device at home between regular clinic visits, and mail blood samples using a private courier such as FedEx on a weekly basis. Participants were instructed to mail devices back regardless of whether the blood collection was successful. The study covered the cost of shipping samples. Participants were instructed to conduct two blood draws, using two devices each time – on one or both upper arms. Because the device was under investigation at the time of the study, viral load test results from the blood collected from the device were not returned to participants in real-time. Instead, participants were only informed of their viral load based on tests conducted at the clinic using routine blood draw collection methods during their regularly scheduled monitoring visits.

### Data analysis

All interviews were professionally transcribed by a company specialized in medical transcriptions. Transcripts were also carefully checked for de-identification prior to analysis. Research team members reviewed all transcripts for accuracy, completeness, and fidelity against the audio recordings. Given the formative nature of the study, we employed conventional content analysis to analyze the qualitative data.^36^ We focused our analysis on inductive reasoning to explore perceptions of and experiences with the device. After conducting three interviews per participant and after two team members independently reviewed all transcripts, we determined that saturation had been reached.^37^

One team member compiled all de-identified text responses into one master file for manual coding. We analyzed data by question blocks, i.e. question by question, to view the range of responses received. After careful refamiliarization with the data and review of the transcripts, we extracted salient quotes and ascribed codes or themes. Two team members double-coded the data and organized text units into emergent themes. The first coder assigned initial codes or themes. The second coder reviewed the resultant codes or themes and made refinements. The codebook was inductive and included code names, descriptions, and examples. Discrepancies were resolved by consensus during virtual team meetings. After identification of the main themes and sub-themes, we summarized findings into narrative descriptions. BEAT-HIV, ATAC and DARE CAB members also reviewed the final codes.

### Ethics statement

The study was approved by both the University of Pennsylvania and the University of North Carolina at Chapel Hill IRBs with written informed consent.

## Results

### Study participants

We recruited 17 participants in the device experience and acceptability component: 15 were cisgender males, 1 cisgender female, and 1 transgender woman. Of those, 10 identified as Black/African American, 2 as White/Caucasian, 3 with more than one race, and 2 as other. Participants were between 31 and 60 years old (mean age: 51 years). Most had a high school education or more. The number of blood collection devices used at home for each participant is listed in Table 1 with a mean of 91.5 devices used at home per participant. We observed an overall 87% success rate, or 13% failure rate, in drawing blood with the device from home.

### Pre-use of the home-based blood collection device for viral load testing

#### Motivations to join home-based blood collection device study

We asked participants to discuss their motivations for joining the device study. Participants explained being referred to the study as part of their parent ATI trial. Most participants mentioned wanting to contribute to science and help the HIV community. Participants understood the device was experimental, and that the device represented a logical next step in how PWH could eventually monitor their viral load as part of ATI trials.

It's a new frontier… If I tell people about the future of blood collection, they wouldn't believe me right now.

– TASSO-8 – Timepoint #2

With the study I'm doing now, it's to find a cure for HIV, and if this helps other people with other health conditions where they don't have to run into a lab all the time to get blood taken… It's something that's going to help people out hopefully, and with the whole COVID thing, it could make life easier where people don't have to run into the doctor's office or the hospital to get labs done all the time.

– TASSO-9 – Timepoint #1

A number of participants noted the convenience of joining the device study because it was embedded as part of the ATI trial. The device provided an alternative way to draw blood for intense viral load monitoring.

I was thinking about the period of time when I would not be in the office maybe the week when I'm not in the clinic for viral load testing… So I just wanted to have extra eyes on my count and I think the Tasso device is perfect for that.

– TASSO-20 – Timepoint #1

Three participants explained being curious about the device as a reason for joining the study. As the study progressed, participants were eager to learn more about the reliability of the viral load test results obtained from the device.

Because I do the regular blood test, but I don't have access to the results gotten from the device. It would be nice to compare and see how reliable they are.

– TASSO-8 – Timepoint #3

### Experiences with using the device

#### Reactions to home-based blood collection device for viral load testing

Participants provided their reactions to the home-based blood collection device throughout the study. Overall, impressions about the device were positive. Some appreciated the novelty and innovativeness of the device.

We could be moving science along enough that people would be able to do blood draws at home, in their own leisure and mail it back to the lab. I thought that was innovative in and of itself.

– TASSO-2 – Timepoint #1

Two participants characterized the device as timely, noting that similar implements have been available for people with diabetes for decades. Several participants noted the convenience of having blood drawn at home. Others appreciated various features of the device.

I liked the smallness of it, the weight of it. I was looking forward to actually getting my hands on it… Seeing how easy it looked and I was like, well, I wish when I was diabetic, I wish my monitor was this small or something I can do just like that. That's what I was wishing.

– TASSO-11 – Timepoint #1

Two participants described how the device would provide additional reassurance during their ATI trial (although they did not receive viral load test results because the assessment of its accuracy or viral load testing is still experimental).

If you're undetectable, [it helps you] make sure you stay undetectable.

– TASSO-8 – Timepoint #3

I guess if the results would come back to undetectable… I guess within that week, that would definitely give someone like me a peace of mind to know that their viral load is managed.

– TASSO-20 – Timepoint #3

Initial negative reactions included needle aversion, skepticism, hesitation, or confusion about the device. These reactions subsided over time after repeated uses.

I couldn't understand the little plastic thing and all of them tubes I was thinking of. No, I didn't think it was going to work.

–TASSO-7 – Timepoint #1

#### Experiences with testing device: perceived ease of use, comfort, safety and pain

Participants described their experiences with testing the device at the three study timepoints, with a focus on perceived ease of use, comfort, safety, and pain.

##### Perceived ease of use.

Most participants reported how easy they found the device to use. This ease was attributed to the clear instructions and participant-friendly test kit. Furthermore, some participants mentioned that the device was easier to use than regular blood draws performed in the clinic.

I think it's very easy to use. It's a lot easier than watching somebody poke you with a needle and having to get the shakes because the size of the needle… It's a lot different than the regular lab sample tests.

– TASSO-2 – Timepoint #3

Most participants mentioned using the device the exact same way each time. One participant (#016) compared the device to a medical prescription.

I think it's easier if you follow exactly as prescribed… It's basically a prescription, so that we can get blood collection.

– TASSO-2 – Timepoint #2

A few participants, however, experimented with the placement of the device to find ideal positioning on the arm. Some participants reported frustrations that the device could only be used on specific body locations. Only one participant tested the device on other parts of the body, such as the upper thigh.

##### Perceived comfort.

Most participants found the device comfortable to use, and this comfort increased over time. Some participants explained how they incorporated the device into their routine.

It's the growing comfortability. And so first I can imagine everyone will be a little nervous the first time, the second time. But the more you do it, the more you're so familiar with it that it's not even a big deal anymore… But once you use it a few times, it's not a big deal anymore… There's a learning curve… After that learning curve and making some mistakes and trial and error… I feel more comfortable with it.

– TASSO-18 – Timepoint #2

I finally figured out exactly where to place the device so that all the little dots fill up fast. Like, you've got a big arm, so you got to find the right spot before you push that button. And now I've got it mastered.

– TASSO-5 – Timepoint #3

Few participants, however, expressed device use fatigue associated with the number of times they were asked to use it during the ATI, when viral load monitoring was occurring in clinic each week –i.e. using 2 devices and weekly home mailing.

##### Perceived safety.

Most participants reported feeling safe using the device. Perceived safety was attributed to the lesser chances of hematomas compared with normal venipunctures, avoidance of blood splatter, limited risk of contamination, small lancet (needle), and limited COVID-19 exposure in the clinic.

Less chances of maybe hematomas and stuff from nurses that don't know what they're doing. No offense.

– TASSO-9 – Timepoint #1

There's no room for error. Unless you lay it down on a dirty surface. It's sort of like wearing a mask. If you lay it down on a dirty surface… you're in trouble.

– TASSO-2 – Timepoint #3

##### Perceived pain.

Participants mentioned minimal pain while using the device. Most described the device as less painful than regular needles. Participants described the device sensation as a ‘pinch’ or ‘dull prick’.

Let's put it this way: This is the least painful way I ever took any kind of needle to get anything done.

– TASSO-14 – Timepoint #2

It's still a dull prick, it's not sharp. So I'm describing it as a dull prick, because it's so faint and so insignificant for that first three seconds, you can feel something metallic touch your skin, but it causes no pain. There is no sharp pain, like the stick of a sewing needle, very, very sharp. But if you stick somebody with the back end, where the thread goes in the needle, that's a dull stick, as opposed to a sharp stick. So the dull stick is really unpainful.

– TASSO-2 – Timepoint #2

Some participants, however, reported residual puncture marks on the arm after repeated use of the device. Some darker skinned participants reported discoloration on their skin from repeated use.

I could see the mark on my arm where it was at, the hole where the needle actually stuck my skin, but other than that I'm not experiencing any type of discomfort, it's not painful.

– TASSO-3 – Timepoint #1

#### Factors that would promote or prompt using device

We asked participants to discuss what helped them use the device. Most found the in-clinic demonstration, video, and instructions most helpful at the first time-point. Participants appreciated being supported by study nurses throughout the study.

[Study member] used it at that medical facility where I see him. It was introduced to me in the medical environment with a medical professional who was excellent at communicating and explaining things. It was introduced to me in a way that was very professional and medical. I think the way it's first introduced to people can really matter, that first impression.

– TASSO-18 – Timepoint #3

At the second and third timepoints, most participants mentioned strategies to enhance blood flow, such as using heat – e.g. heat pads or hand warmers to warm the area where the device will be placed and/or applying the device right after coming out of the shower.

I usually wait until I get out of the shower, so this way my arms getting warmed up and then I know the surface is clean.

– TASSO-9 – Timepoint #2

I thought that was a fabulous idea to put the hand warmer packs in there.

– TASSO-11 – Timepoint #2

Additional blood flow techniques included massaging, rubbing, or flexing the arm prior to using the device.

I put the heating things on it, because they just started putting hand-warmers in, to warm up the space. But I also find that doing a little isometric pumps with your arm, kind of get blood flow in my arm faster than rubbing it or putting the heating hand-warmer on it.

– TASSO-2 – Timepoint #2

Additional factors that would help using the device included using a mirror, good hydration, and avoiding lotion on the arm to ensure adherence of the device. All participants reported being able to use the device by themselves without assistance.

#### Difficulties with using the device

Participants reported some difficulties with using the device at each of the three timepoints. The most prevalent issue was the failure of some devices to completely fill and blood coagulating too soon. As a result, participants felt that they did not complete the task or did something wrong.

It is nervewracking that all the time that all the chambers don't get filled. I think it's only happened twice, never at the same time, if I've not gotten a blood draw. I've always at least had one… So I think if, when you're doing it, and I knew I was placing them in the correct place… He [study nurse] had to remind me that it's just a prototype and we're trying to work out kinks.

– TASSO-11 – Timepoint #3

The second most common difficulty was the device not sticking to the arm. In some cases, the device fell off and participants were unable to reuse it, or the device falling resulted in a minor blood spillage.

It bothered me that once you stuck it in, once you press that needle out and it does fall out of your arm, there's no reusing it. So I felt bad that once it fell out that it was no longer usable. I wish that once you press that needle, it's a one and done thing.

– TASSO-18 – Timepoint #2

One participant reported difficulty with removing the device.

I guess the only difficult part is when you're taking it off. Because I guess because of the glue that's on it. You got to pull it, you got to pull it off. So that was the only thing. But other than that, wasn't bad.

– TASSO-1 – Timepoint #1

Additional issues encountered included trouble remembering to detach the sticker prior to use, a second mysterious click after the initial push (possibly due to the needle retracting), trouble remembering to write the date and time of blood collection, and the inconvenience of having to wear a band-aid or irritation from it. One participant could only use the device on the left arm due to a history of blood clot on the right arm.

I can only use it on the one arm because I have a history of blood clots in my other arm… We've been just using my left arm for everything… My arms aren't that wide, so I usually just put one up a little higher than the other.

– TASSO-9 – Timepoint #3

#### Perceived advantages of the device

Participants described several advantages of the device. The most common perceived advantage was the convenience it would provide, such as reducing the need for frequent clinical monitoring visits during ATIs.

I have to go about eight miles to my doctor. And because I catch public transportation, that's an event. That's four hours. Two hours there and two hours back. I'm six minutes doing the device and probably another eight minutes walking to the drop off. If I wanted to catch the bus there, I could. But it's convenient.

– TASSO-11 – Timepoint #2

The device was found helpful during the COVID-19 pandemic, and conferred control over one’s health care and was perceived as empowering.

It's part of us taking control of things. And in these years… I've noticed the people who really become informed and educated, and take control of their health and their wellbeing. Not just their health, but emotionally and physically, and all that stuff. They're the ones who sort of become empowered and take control, and have control of their health… It's part of giving us back our independence that we kind of couldn't lose with a lot of our health concerns or we're feeling consumed by them, or stigmatized by them. This gives us back a lot of power and control, I think. And that can lead to empowering us and just giving us a lot of… giving back a lot of that self worth that we kind of can move.

– TASSO-18 – Timepoint #1

Other perceived advantages of the device included confidentiality, obtaining faster medical information, and no need for venipuncture or large needles. The device configuration alleviated the worry of having to poke oneself.

That's what it is. I don't see the needle, so it happens and then it's done. I don't have to worry about seeing myself poking myself.

– TASSO-20 – Timepoint #2

One participant described how his anxiety grew during the ATI, and how the device could help alleviate the worry of being off ART.

Whatever feelings I went into this with, have gradually changed since I've been on it [the ATI]. I think I told I was not anxious to stop my ART, but now that I have, it's been a change. I've noticed a change in the way I view it all. And so again, with this here, it's less trips to the doctor. I can see that now.

– TASSO-18 – Timepoint #2

If proven effective, the device would eventually provide a benefit to society and individual patients/participants. PWH envisioned how the home-based blood collection device could keep on evolving.

I do believe that the longer we continue to use it… I believe that with all the information, that data, that you're getting, that it's going to get easier for us and y'all will come up with some solutions that'll make it even easier for us.

– TASSO-1 – Timepoint #2

#### Perceived concerns about the device

Participants expressed limited concerns about the device across the three timepoints. Perceived concerns included the need for transportation to mail in samples and the possibility of developing a dependence on the device for blood draws. Another participant was anxious about packing the device kit correctly before mailing.

I guess taking everything out of the package and lay everything out. That's the easiest. The hardest it's the opposite. When you got to pack everything back up and make sure you put dates on this and dates on that. And this is sealed. You want to make sure that it's nice and tight.

– TASSO-6 – Timepoint #6

One participant wanted to be re-assured that there would be no major issue with his viral load while using the device.

I wanted to take the test and not hear anything from anybody and once later find out, Oh, well, your viral load is out of control. It's been out of control. You should have done something about this way back when."

– TASSO-12 – Timepoint #1

Participants suggested possible contraindications to using the device, such as hand coordination or dexterity issues, arthritis, fibromyalgia, hemophilia, or hypersensitivity.

It doesn’t work well for someone who may have arthritis, they can't get the grip strength to push the red button. But other than that it's very, very easy.

– TASSO-4 – Timepoint #2

It has no problem and it should be very easy, it is a very, very dull prick for anybody that gets it, I would imagine, unless they're hypersensitive and have fibromyalgia and really hypersensitive nerve endings.

– TASSO-2 – Timepoint #2

#### Suggestions to improve the device

We solicited suggestions to improve the device at timepoints 2 and 3. Suggestions pertained to the device mechanism or placement, device features and the device kit.

In terms of the device mechanism, some participants suggested enhancing suction or having a longer lancet to facilitate blood flow, combined with ways to prevent blood clotting or coagulation. Others wanted to find a different placement instead of the upper arm so the device would be easier to see during blood filling, or where capillaries would be more accessible.

I think they're just relying on gravity and it's not enough actually… If there was a way to do that in the main part of where the little sticky thing happens, the little lancet, and then that little area with the red button has a way of having a vacuum or a suction, like a quick little amount of suction that would help to actually draw the blood and put it in the paper chambers.

– TASSO-4 – Timepoint #3

It's going to be on the side of your arm, unless they could change the placement of it where you could see it easier without looking in the mirror or trying to bend your neck like a giraffe to look at it.

– TASSO-10 – Timepoint #2

Improved device features would include a smaller or narrower device, stronger adhesive, and allowing participants to write the date and time directly on the device instead of using labels. Two participants mentioned changing the device color, because the current red color may be triggering or associated with pain.

If people need instead of a red push button, if it had blue push buttons or yellow, or something that brings people comfort, it should be assorted colors available, because it gives people a connection to the device. People connect with things very, very oddly. We have different styles of learning, so it's just one of those things that can be encouraging to a person, if they had different push points, that little red button. Yeah, I think that would be helpful for someone in the future, but not necessarily me.

– TASSO-2 – Timepoint #3

Suggestions to refine the device kit included larger alcohol pads, better quality band-aids, a viral load journal or diary, a technical contact in case of questions and a warning that the device is not a toy for children.

#### Suggestions to improve the device research experience

Most participants had no suggestion to improve the device research experience.

There isn’t anything. It's such a simple solution to drawing the blood… It's pretty cut and dried.

– TASSO-10 – Timepoint #2

Four concrete suggestions were offered to improve the overall research experience: providing more devices to practice or additional back-up devices, sending texts reminders to remind participants when to use the device, being able to mail device kits from home, and providing geographical maps of nearby private couriers for mailing blood to the laboratory. Some of these suggestions were implemented during the study.

### Experiences with mailing blood samples

Participants reported overall positive experiences with mailing blood samples and encountered no issue with same-day shipments. This was due to the wide availability of private couriers in the city (Philadelphia), and the pre-labeled and pre-paid device kit.

Every thing about this thing is easy, because if it wasn't, I wouldn't do it!

– TASSO-7 – Timepoint #2

No, because it's a self-addressed, stamped, envelope, postage paid.

– TASSO-2 – Timepoint #2

Participants expressed trust in the private courier and no major fear with mailing blood. One participant would be apprehensive using the U.S. Postal Service to mail blood.

If I had to use the United States Post Office, that would definitely cause me a problem. It would actually make me not do it.

– TASSO-5 – Timepoint #1

Reported challenges with mailing blood samples were remembering to mail the package, finding a drop-off location, and missing the evening drop off time and not having a weekend pick-up.

Well, at first was locating a box because I've never used FedEx before. I've never mailed anything FedEx. So I just wasn't familiar with that process. And now that I know where a box close by is, it's just a matter of remembering to put the package in my vehicle and remembering to stop as I pass by here.

– TASSO-12 – Timepoint #2

### Communicating viral load test results and desired sensitivity

Participants shared their preferences for communicating future viral load test results when viral load measures will be available in real-time. Most participants preferred a web portal or an app that would notify them of their viral load test results in real-time. Others mentioned emails with secure attachments or phone calls. A few participants mentioned having access to a doctor to receive a consultation if needed or utilizing telemedicine which has increased in popularity since the COVID-19 pandemic.

I have an app on my phone called MyPenn. You can just open it up on your phone and it gives you all your medications and your everything, your backups, your medication, your readings, and vitals or whatever the case may be.

– TASSO-15 – Timepoint #3

I think what we managed to do is the telemedicine, that whole transition piece, because now most places for healthcare has a portal you can log into and look at your results, look at any outstanding messages or any urgent messages, because they're indicators… There's nothing more, because we're already fully transitioning.

– TASSO-8 – Timepoint #3

In terms of the sensitivity of the viral load test, most participants wanted results to be as accurate as possible, because health decisions would be based on those results. One participant wanted the viral load test to be sensitive enough to determine when PWH became detectable to self-assess whether they could pass on HIV to sex partners during the ATI.

Well, I mean, obviously, it should be true viral loads. If they're getting CD4s, whatever, I don't know all that's going to be included in this blood work that these will be doing, but yeah, they have to be accurate because you're going to be basing on all your health decisions on that.

– TASSO-10 – Timepoint #3

It needs to be especially good. If not only me, I know, people meet people and they want to be honest with people and it's showing them immediate results and showing them documents that show that you're undetectable and your CD4 is high, which means you don't have the virus to spread. You're undetectable, meaning you're untransmittable. That would really, really take away so many peoples… I know people who live very solitary lives, lonely lives, because they're afraid of spreading it or afraid of disclosing. This will transform people's fear.

– TASSO-18 – Timepoint #3

### Device use beyond ATIs

#### Perceived importance of the device and testing viral load from home

Most participants would see themselves using the device on a regular basis outside of the ATI context. By timepoint 3, most participants had developed self-efficacy using the device and would like it to be part of regular HIV care. One participant was ‘awaiting [his] prescription’ for the device. Another participant expressed wanting the device to be covered by health insurance.

Now that I'm comfortable with it and familiar with it and I've grown comfortable with it from using it. Yes. It's a learning curve, so everyone's going to be awkward and uncomfortable in the beginning, which I was too. But now I feel very competent with it.

– TASSO-18 – Timepoint #3

In terms of the perceived importance of being able to self-test viral loads from home, participants viewed the device as an additional source of health information, as well as an opportunity to ask questions about their virus. Two participants, however, noted the critical role of providers in helping them interpret viral load numbers. One participant preferred the full blood screening offered during regular clinic visits, instead of focusing solely on the viral load.

That's for the doctors. I mean, I'm serious. That's what the doctor's asking for, so that should go to them. Because you give me the test, I'm looking at it, I don't know what I'm looking at. I just see a whole bunch of numbers, and whatever.

– TASSO-7 – Timepoint #3

I understand the importance of doing the viral load, but I also want a full review of where I'm at with my health.

– TASSO-20 – Timepoint #3

One participant would still want interactions with health care workers in the clinic and acknowledged the critical role of psychosocial support during clinic visits. This participant cautioned that the device could further increase isolation for PWH.

It's the way that science is going. That's the way life is changing. I would still rather go to the doctor's office, talk to the [nurses], ‘Hey, how you doing? … Y'all have a good one.’ That routine. That's the part I'm going to miss or would miss about it. The personal part of healthcare. The thing that kind of made this disease bearable in the beginning. They let me know I wasn't alone, and I had people if I needed them that knew what I was going through. But if I don't have to do that in the office, if it takes away from going into the office, it reduces my contact with people that I know understand the life I have to live. That's just the way I think…. It's a great device, but for certain diseases, especially where there's a psychological component to it, that loneliness or not being able to go to the office, I didn't miss it until COVID kicked in. So now I can really appreciate it. The device would only further keep us isolated away from each other. So again, that's just the way we're probably going with science or we would have to just deal with it, but I'd rather not… Great. But in the city, too much depression. You need to go see the doctor.

– TASSO-5 – Timepoint #3

#### Possible effects on stigma and social risks

We asked participants about potential social risks of the device and the effects of the stigma at the three timepoints. Most described how the device would have a positive effect on internal and external stigma, by normalizing HIV as a chronic condition, reducing doctor’s visits, and helping with disclosure of undetectable HIV status.

This [device] helps with stigmatism, stereotypes, no one has to see you going to the office to go get blood work done.

– TASSO-11 – Timepoint #2

For people who don't really understand anything about this disease, if they had the understanding that a person like myself who is undetectable and has been undetectable since I was diagnosed, that if they can see or get an understanding of this little bit of blood right here shows that there is not enough copy of the virus in my system to transmit it to other people, they would get a better understanding of how you don't have to be afraid of who I am just because I've been diagnosed with HIV. There's nothing to be afraid of.

– TASSO-3 – Timepoint #2

The residual marks on the skin or discoloration from repeated use of the device may cause stigma. Further, a few participants did not see how the device would help reduce HIV-related stigma because stigma remains pervasive in society.

I believe that the stigma associated with HIV is so ingrained in society now. They don't think about it much, but it's always there. I think it will always be there.

– TASSO-5 – Timepoint #2

One participant noted that the frequent visits to FedEx could identify him as living with HIV. This participant took the additional precaution of hiding the device kit in a bag during the study.

After a while I started to feel like I should be more discreet and not just carry the bag in the open, but that again is a personal preference. Now I put it into a bag, so no one can see what it is from just seeing me walking with it.

– TASSO-18 – Timepoint #3

Yet several participants applauded the fact that the device kit contained no HIV-specific information, and noted no social risk associated with using the device.

It's just a device for collection of blood. We are testing this in a specific population, but it doesn't say. It has no indicator that it's for people living with this or that… I don't think it's a threat to anybody's security about their status.

– TASSO-2 – Timepoint #1

#### Additional potential uses for the device

Participants envisioned additional potential uses for the device besides viral load testing. For example, they would like the blood to be able to measure CD4 or CD8 and other lab values, such as chemistries. One participant would prefer if the device could take care of all the lab work simultaneously.

Probably could take care of all my blood work in that one dose.

– TASSO-14– Timepoint #2

Participants mentioned other health conditions that may benefit besides HIV, such as diabetes or any other illness requiring frequent blood draws.

I didn't see it as an HIV tool, I see it as something that's progressively can be expanded to assist other people with illnesses.

– TASSO-2 – Timepoint #2

I think this, because it draws blood from intramuscularly as opposed to intravenously, I think it will serve, over the next 10 years, we could find a broader-based use if medical science wants to find and utilize this in a broader way. It is already a propos to other domains and fields of study.

– TASSO-2 – Timepoint #3

Additional groups that may benefit from the device included those who are a hard stick or those with small veins, lost to care, in nursing homes, with mobility issues or living in rural areas.

I've had the experience of dealing with people who are lost to care and by making it easier to get the sample, to help them understand that they [need] to use the meds, they need to do what they got to do.

– TASSO-4 – Timepoint #2

People who aren't mobile or people who live in rural areas, I could see this being a great assistance for all types of illnesses, not just HIV.

– TASSO-18 – Timepoint #3

Further, one participant proposed the device be used for drug screening. Overall, pluri-potency of the device – or the ability to conduct multiple tests and serve diverse populations – would augment acceptability.

#### Additional considerations

Participants were optimistic that the device would become available to facilitate the conduct of ATI trials. They mentioned the device could become an important instrument to give ATI participants peace of mind about their viral loads.

I think it would be extremely helpful… It would be helpful for the providers or the research coordinators and assistants as well as the patients because you collecting the information is great, but it's my health. And for me to stay on top of it, knowledge is power. It would give me that peace of mind.

– TASSO-20 – Timepoint #2

One participant suggested involving PWH as peer educators during ATI trials. Another participant hoped the device would become available to everyone who needs blood drawn, not just PWH.

I'm hoping that it goes well and this one day will be the way people get their blood taken.

– TASSO-10 – Timepoint #2

**Figure 2:**
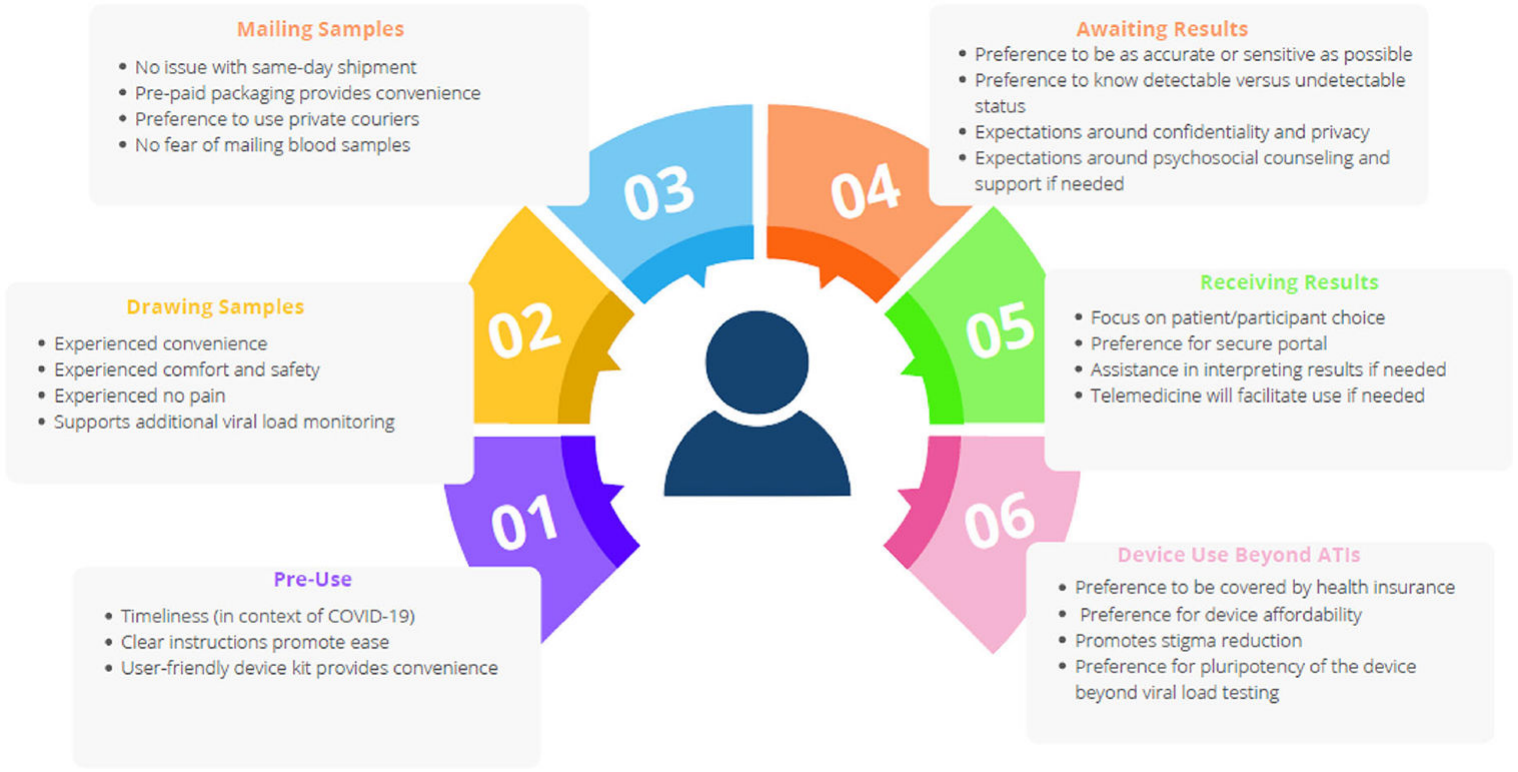
Summary of ATI participant experiences with home-based blood collection device for viral load testing.

Figure 2 summarizes the ATI participant experiences with the home-based blood collection device for viral load testing. Supplementary Appendix 2 includes additional quotes relevant to the above findings.

## Discussion

We found high acceptance of a novel home-based peripheral blood collection device for HIV viral load testing in the context of two cure-related trials including an ATI period conducted in Philadelphia, PA, USA. Most PWH in our study viewed the device as relatively convenient, painless, easy to use, and a simple solution to frequent blood draws. The main challenge encountered was the inability to completely fill up devices with blood in some cases. Most participants reported positive experiences with mailing blood samples and could see themselves using the device on a regular basis outside of an ATI period. Our findings corroborate those of a prior survey which found high hypothetical acceptability of the device in the context of cure-related trials including an ATI period in the United States among PWH, biomedical HIV cure researchers and HIV care providers.^35^ The main strength of the present study was that findings were based on actual experiences with testing the device as opposed to a theoretical scenario.

The current study corroborates several findings from our prior hypothetical acceptability survey in the U.S.^35^ For example, participants validated perceived benefits regarding the convenience, sense of control, lowered anxiety during ATIs, and usefulness during the COVID-19 pandemic. However, our study uncovered several new issues that can only be learned from direct experience. These included issues with the device not filling up properly, imperfect adherence to the arm, concerns with correctly packing the kit before mailing, desire for improved accessories in the device kit, and limited actual issues reported with mailing of blood samples.

Further, the device was perceived as simplifying blood draws and could provide additional reassurance or peace of mind during an ATI period about one’s viral load (un)detectability status. This finding has important implications, because knowing when one has become viremic in the context of an ongoing cure-related trials including an ATI period could help reduce risk of unintended HIV transmission to sex partners.^38^ In the present study, however, PWH did not receive viral load test results from the blood collected by the experimental device but did receive this information from regular draws in clinic visits. If the device proves effective and yields accurate viral load test results, acceptability and uptake will likely increase. Participants wanted the viral loads to be as sensitive as possible and were eager to see future data with side-by-side comparisons of their viral load tests from the clinic versus those obtained from the device to draw conclusions around the device’s effectiveness. Our team is currently analyzing viral loads obtained from the device and will report the reliability results in a separate manuscript. Because our study did not return viral load test results, potential concerns about test accuracy and how this may affect device acceptability remains under-explored,^22,24,25,27^ and should be a topic of inquiry for further research. Participants did comment on preferences for the process of returning detectable viral load test results from devices during ATI periods while retaining a degree of physician consultation. Additional counseling will still need to take place around the need for barrier protection and safe sex practices.^38^

During the study, participants’ perceptions matured from being theoretical to being practical after direct experience with the device. A key advantage of the Tasso home-based blood collection device was the lack of venipunctures needed to draw blood. Similarly, in prior studies, investigators found greater acceptability of blood collection methods that obviated the need to puncture veins (e.g., dried blood spots). These methods are less invasive and have lower risk of soft tissue injury.^39,40^ A dried blood spot experience study conducted in the context of doping control for athletes in Denmark similarly found high usability and limited painfulness of blood collection from the upper arm.^41^ In our acceptability study, participants appreciated the configuration of the device, which alleviated worry around having to poke oneself because the lancet was hidden. Several participants appreciated the sense of control and compared the device with home-based tests employed for diabetes. Unlike the diabetes or pregnancy test, however, device users would still need to rely on mail specimens to an external laboratory for viral load measurements.

Nevertheless, our study uncovered noteworthy concerns with the device. For example, some participants reported initial hesitancy or confusion when first starting to use the device. These apprehensions subsided over time as personal experience, expertise, and comfort with self-collection of blood developed. Support from study coordinators during this initial period was important to retain participants into the device study. Further, participants reported at least some devices not filling up completely, and felt they were improperly completing the task, and wondered if the blood collected was sufficient to measure viral load. The worry of not getting a sufficient or good enough sample seems prominent in the biospecimen self-collection literature.^17^ Our findings are also consistent with those of a separate study using the Tasso device to test for anti-SARS-CoV-2 antibodies, which reported 93% successful unsupervised blood collection.^42^ Similar to the SARS-CoV-2 study,^42^ no participant in our study was unsuccessful with the Tasso self-collection, and we noted an overall 87% success rate in drawing blood from home. However, some participants reported residual puncture marks from using the device, and these could become stigmatizing for PWH. Further, one participant cautioned that some PWH could develop a dependence on the device for blood draws, instead of regular needles. The above concerns should be taken into consideration because they could eventually affect downstream implementation of home-based blood collection as part of cure-related trials including an ATI period.

We received thoughtful suggestions to improve the device. These included refining the device mechanism to ensure sufficient filling and to prevent blood coagulation or smaller device features. While participants appreciated the thoughtfulness that went into assembling the device kit, they suggested some improvements. Participants reported positive experiences with mailing blood samples, mainly due to the pre-paid return boxes and high availability of private couriers (e.g. FedEx) in Philadelphia. Our prior nationwide survey likewise revealed a strong preference for private couriers for mailing in samples, instead of the U.S. Postal Office,^35^ due to trust that specimens could be reliably tracked. However, individuals who live further away from private couriers may need to contend with transportation issues or other barriers that should be considered when rolling out the device.

In terms of communicating viral load test results, participants in our study preferred the user-friendly web portal, combined with other approaches such as email or phone based on their preferences. The recommendation to use telemedicine was notable, since remote physician consultations have gained in prominence during the COVID-19 pandemic and could greatly alleviate participant burden and anxiety. However, technology barriers such as access to computer or internet would need to be taken into consideration if telehealth were to benefit all PWH equitably. In the context of ATI trials, participants would still need to adhere to an in-clinic visit schedule to allow precise measurement of the estimated time to virologic rebound^1^ and to monitor other lab values. While PWH appreciate the convenience of remote viral load testing, they still benefit from interactions with health care staff, together with the psychosocial support offered during clinic visits that guards against social isolation, particularly during the COVID-19 pandemic. It appears that the device could never replace the highly dedicated health care teams involved in supporting ATI trial participants.

Moreover, several participants noted that the device could help normalize HIV as a chronic condition, like diabetes. Participants appreciated that the device kit contained no HIV identifier, and therefore would not represent an intended disclosure threat. However, several participants commented that stigma remains a pervasive reality in their lives. This was evidenced by one of our study participants taking additional precautions to hide the device kit. More research will be needed to understand the social consequences of using home-based blood collection for HIV viral load testing in diverse settings, and how this would affect both internal and external stigma for PWH, and help to identify possible ways to mitigate stigma.

Overall, study participants were hopeful that the device could become the new reality of blood collection for PWH. They were also optimistic that individuals with other illnesses may also soon benefit from this blood draw device. Augmenting pluri-potency of the device to encompass other tests besides viral load may also increase acceptability. The World Health Organization has issued ASSURED criteria to guide acceptability of point-of-care diagnostics, such as, affordable, sensitive, specific, user-friendly, robust, equipment-free, deliverable.^11^ These criteria would be applicable to all possible tests conducted with the peripheral blood collection device. Further, implementation science may become necessary to optimize scale-up in various settings.^11,28^

### Limitations

We acknowledge limitations to our study. We interviewed a small number of trial participants at one location (Philadelphia, PA, USA), and results may not be generalizable. Although our sample was diverse with respect to race and ethnicity, it was mostly composed of cisgender men. Thus, we were unable to detect differences by sex or gender. To assess acceptability of the device on a broader scale, researchers will need to test as part of larger cohorts of participants. Our study was conducted in the context of ongoing cure-directed trials including an ATI period with regular visits and weekly interactions with participants, which may have skewed responses towards greater acceptability of home-based tests due to the added study support. A major limitation of our study design was the separation of the acceptability and reliability components of device testing because we did not return viral load test results from the device to the participants in real-time. We expect that acceptability of the device would increase with greater access to results in real-time and confirmed accuracy of the viral load tests. We did not interview participants who declined participation, and therefore we may remain unaware of potential barriers to device uptake. All participants resided in an urban setting with access to private couriers. More attention will need to be paid to barriers of returning self-collected samples, particularly for those who live in rural areas or with transportation limitations. These limitations notwithstanding, a major strength of our study was the richness of the data generated around acceptability of the Tasso device during ongoing cure-directed trials including an ATI period. We reached data saturation,^37^ and presented findings with fidelity to the data.

## Conclusions

Our study showed acceptability of a novel home-based peripheral blood collection for viral load testing in the context of two cure-related trials including an ATI period implemented by the BEAT-HIV Martin Delaney Collaboratory. Participants recognized the device as experimental and were eager to see further refinements materialize to facilitate self-collection of blood. Measuring reliability of viral load tests is a significant requisite next step and will be reported separately. We expect that the ultimate opinion of users on the value of the device will depend on how well it can measure viral load and whether this information will be available to them in real time. If blood collected from the device is found to accurately measure viral loads in the separate reliability component of the study, the device could prove a critical adjunct to ongoing HIV cure-related clinical trials and significantly reduce the burden for participant of trips to the clinic to monitor viral load changes during ATIs periods. More research will also be needed to optimize interventions aimed at reducing the anxiety of being off ART and developing patient/participant-centric strategies in the context of ATI trials requiring prolonged viremia. The COVID-19 pandemic provides another critical inflexion point to devise more convenient ways to remotely self-monitor one’s health. If clinical trial results are positive, hopefully this device will evolve to benefit other fields of research and care beyond HIV – to include the management of other chronic illnesses..

## Supplementary Material

Supplemental Appendix 1

Supplemental Appendix 2

## Figures and Tables

**Table 1:** Demographic characteristics of study participants (Philadelphia, PA, United States, 2021–2022).

Tasso participant ID	Gender	Sex assigned at birth	Age	Race	Ethnicity	Education	Timepoint #1	Timepoint #2	Timepoint #3	# At-home blood collections	Comment
Participants from ‘*A Phase I Study to Evaluate Innate Activation Predictors of Sustained Viral Control in Adults Living with HIV Undergoing a Brief Analytical Treatment Interruption after the Administration of Pegylated Interferon Alpha 2b in Combination with Two Intravenous Broadly HIV-1 Neutralizing Antibodies 3BNC117 and 10-1074*’ (NCT03588715) (Penn Presbyterian Medical Center)											
TASSO-1	M	M	59	Black/African American	Non-Hispanic/Latino	Some College	January 2021	March 2021	September 2021	118	Post-Intervention Controller
TASSO-2	M	M	58	Black/African American	Non-Hispanic/Latino	2-year degree	January 2021	March 2021	July 2021	92	
TASSO-7	M	M	45	Black/African American	Non-Hispanic/Latino	High School Diploma	January 2021	March 2021	April 2021	47	
TASSO-8	M	M	31	Other	Hispanic/Latino	2-year degree	February 2021	April 2021	February 2022	164	
TASSO-9	M	M	M	More than One Race	Non-Hispanic/Latino	Some College	January 2021	March 2021	October 2021	118	
TASSO-10	M	M	60	White Caucasian	Non-Hispanic/Latino	High School Diploma	January 2021	March 2021	July 2021	94	
TASSO-11	M	M	56	Black/African American	Non-Hispanic/Latino	Some College	January 2021	March 2021	September 2021	122	
TASSO-18	M	M	49	More than One Race	Non-Hispanic/Latino	Some College	May 2021	June 2021	July 2021	58	
Participants from ‘*A Phase I Study to Evaluate Innate Activation Predictors of Sustained Viral Control in Adults Living with HIV Undergoing a Brief Analytical Treatment Interruption after the Administration of Pegylated Interferon Alpha 2 b in Combination with Two Intravenous Broadly HIV-1 Neutralizing Antibodies 3BNC117 and 10-1074*’ (NCT03588715) (Philadelphia FIGHT Community Health Centers)											
TASSO-3	W	F	52	Black/African American	Non-Hispanic/Latino	High School Diploma	February 2021	March 2021	November 2021	150	
TASSO-4	M	M	52	More than One Race	Non-Hispanic/Latino	2-year degree	January 2021	March 2021	June 2021	86	
TASSO-5	M	M	51	Black/African American	Non-Hispanic/Latino	4-year degree	January 2021	March 2021	August 2021	84	
TASSO-6	TW	M	54	Black/African-American	Hispanic/Latino	High School Diploma	February 2021	March 2021	July 2021	76	
TASSO-20	M	M	37	Black/African American	Non-Hispanic/Latino	High School Diploma	June 2021	July 2021	February 2022	108	
Participants from ‘*CD4 CAR + Zinc Finger Nuclease (ZFN)-modified T Cells in HIV Therapy*’ (NCT03617198) (Hospital of the University of Pennsylvania)											
TASSO-12	M	M	60	White/Caucasian	Non-Hispanic/Latino	2-year degree	January 2021	March 2021	February 2022	100	Post-Intervention Controller
TASSO-13	M	M	39	Black/African American	Non-Hispanic/Latino	Some High School/No Diploma	March 2021	April 2021	August 2021	69	
TASSO-14	M	M	48	Other	Hispanic/Latino	2-year degree	February 2021	March 2021	Missed Window	8	
TASSO-15	M	M	56	Black/African American	Non-Hispanic/Latino	4-year degree	January 2021	June 2021	October 2021	61	

M = man/male; F = female; TW = transgender woman.

## Data Availability

All data relevant to this study have been provided in the text and in the Supplementary Appendices.
