# Supplementary material for: Participant experiences using novel home-based blood collection device for viral load testing in the HIV cure trials with analytical treatment interruptions
Source: HIV Res Clin Pract. Author manuscript; Available in PMC 2022 Aug 25. (PMC9403870)
Supplement: Supplemental Appendix 1 [file NIHMS1830173-supplement-Supplemental_Appendix_1.docx]

**Supplementary Appendix 1: Interview Guides for Participant Experiences using Novel Home-Based Blood Collection Device for Viral Load Testing in the Context of HIV Cure-Related Research with ATIs**

**(Philadelphia, United States, 2021)**

| **Timepoint #1**   - What do you think of the home-based viral load testing device?   - What were the pros? What were the cons? - How did you make the decision whether to join the home-based viral load testing acceptability study?   **Questions for Accepters**:   - What are some of the reasons you decided to participate in the home-based viral load testing study?   - What was your strongest motivation?   - Do you think the home-based viral load test device will help you meet any needs that you have?     - If so, what are they? - What do you think it will be like to use the device at home?   - Do you feel comfortable using it?   - Do you think it will be safe?   - Do you think it will be painful? - What sorts of things would make it easier for you to use the device? - Do you think you will have any difficult using the device?   - [Additional prompt: Please describe] - Are you excited about testing the device? If so, what do you find exciting? - Are you worried about testing the device? If so, what are you worried about?   - Prompts:     - Do you have concerns around mailing in your samples?     - Do you have concerns that the device may identify you as a person living with HIV (e.g. people may recognize that you are living with HIV if you use the device)?     - Other concerns? - Do you have any questions about the device?   **Questions for Decliners**:   - What is the main reason you decided not to participate in the home-based viral load test study? |
| --- |
| **Timepoint #2**   - How is the HIV treatment interruption going? - At this point in the study, what are your reactions to the home-based viral load test device?   - Good reactions? Bad reactions? - What has it been like for you using the device at home?   - Did you feel comfortable using it? Has it become more comfortable over time? In what ways?   - Did you find it easy to use? Did you use the device in the same way each time?   - Do you think it is safe? Please explain.   - Do you think it is painful? Please explain. - What helped you use the device? - Was it necessary for anyone to help you use the device? - Have you had any difficulties using the device?   - If yes, what were some of the difficulties? - Would you change anything about the device?   - If so, what would you change?   - Do you have any suggestions to improve the device? - What could the research team do to help improve your experience with the device? - Where there any issues with the logistics of mailing in your samples?   - Did you have any problems mailing in your samples?   - What has been helpful when mailing in your samples?   - Did you have a problem mailing samples on the same day?   - How far did you have to go to mail in your samples? - What do you like most about the device?   - What do you find most easy?   - Are you excited you about continuing to use the device? - Do you have any worries about continuing to use the device?   - If so, what are some of your worries?   - Did you have concerns that the device may identify you as a person living with HIV (e.g. people may recognize that you are living with HIV if you use the device)? - Do you think that being able to test your viral load at home would help reduce stigma around HIV? If so, how? - At this time in the study, do you have any questions about the device? - Is there anything else you would like us to know about the device? |
| **Timepoint #3**   - Did you have any problems with your HIV treatment interruption go? - At this point in the study, what are your reactions to the home-based viral load test device?   - Good reactions? Bad reactions? - What has it been like for you using the device at home?   - Can you walk me through the last time you used the device?   - Did you feel comfortable using it? Has it become more comfortable over time? In what ways?   - Did you find it easy to use? Did you use the device in the same way each time?   - Do you think it is safe? Please explain.   - Do you think it is painful? Please explain. - What helped you use the device? - Was it necessary for anyone to help you use the device? - Have you had any difficulties using the device?   - If yes, what were some of the difficulties? - Did anything happen to you while you were using the device?   - If so, can you please describe? - Would you change anything about the device?   - If so, what would you change?   - Do you have any suggestions to improve the device? - What could the research team do to help improve your experience with the device? - Where there any issues with the logistics of mailing in your samples?   - Did you have any problems mailing in your samples?   - What has been helpful when mailing in your samples?   - Did you have a problem mailing samples on the same day?   - How far did you have to go to mail in your samples? - What did you like most about the device?   - What did you find most easy?   - What excited you about using the device? - Did you have any worries about using the device?   - If so, what were some of your worries?   - Did you have concerns that the device may identify you as a person living with HIV (e.g. people may recognize that you are living with HIV if you use the device)? - If the home-based viral load test device proves effective, what would be the best way to communicate test results to you?   - Additional prompts: In person by your doctor? Telemedicine? Over the phone? Email? Postal mail?   - How sensitive (or how good) do you think the viral load test result should be? - Would you see yourself using this device on a regular basis?   - If yes, why?   - If no, why not? - Do you think it is important to be able to test your viral load at home?   - How so? - Do you think that being able to test your viral load at home would help reduce stigma around HIV? If so, how? - Do you think there might be other uses for the home-based HIV viral load test outside of HIV cure-directed clinical trials involving ATIs?   - [For example, person living with HIV not involved in HIV cure-directed research] - At this time in the study, do you have any questions about the device? - Is there anything else you would like us to know about the device? |
