# Supplementary material for: Participant experiences using novel home-based blood collection device for viral load testing in the HIV cure trials with analytical treatment interruptions
Source: HIV Res Clin Pract. Author manuscript; Available in PMC 2022 Aug 25. (PMC9403870)
Supplement: Supplemental Appendix 2 [file NIHMS1830173-supplement-Supplemental_Appendix_2.docx]

**Supplementary Appendix 2: Additional Quotes for Participant Experiences using Novel Home-Based Blood Collection Device for Viral Load Testing in the Context of HIV Cure-Related Research with ATIs**

**(Philadelphia, United States, 2021)**

| **Themes and Sub-Themes** | | **Participant Number** | **Quotes** |
| --- | --- | --- | --- |
| **Pre-Use of the Home-Based Blood Collection Device for Viral Load Testing** | | | |
| **Motivations to Join Home-Based Blood Collection Device Study** | | | |
| Referred by parent trial or doctor | | TASSO-5 – Timepoint #1 | *Because my doctor recommended it… If he suggests something, or if he says something new was out there, he's not going to suggest anything that's not safe… I trust him with my life... So he mentioned it and I said, "I'm on board, not a problem.”* |
|  |  | TASSO-12 – Timepoint #1 | *My doctor asked me to.* |
| Contributing to science | Scientific progress | TASSO-10 – Timepoint #1 | *I pretty much say yes to every study… I'm a human pin cushion.* |
|  |  | TASSO-11 – Timepoint #1 | *Mind you, when I first started taking medicine, I was taking upwards to 27 pills a day, but they wasn't all HIV related. I was taking seven pills with that, but I take one pill. But now, I'm not taking any HIV medicine.* |
|  |  | TASSO-2 – Timepoint #1 | *It was a quick decision, because again, it was talking about progress. Just thinking like somebody that's convalescent at home, and a lot of people have especially seen, they have home health aides. To find a way that they participate in getting blood drawn without having to leave their home, that whole convenience component made it real easy to want to test and see what was on the other side.* |
|  |  | TASSO-2 – Timepoint #2 | *And I think this is vanguard, avant garde.* |
|  |  | TASSO-4 – Timepoint #1 | *I have been doing a lot of research projects and studies before, so I am all for the science. And it was like, so, explain to me how this works and what's it for… And I'm like, yeah I'll do it… I'm a part of science history.* |
|  |  | TASSO-6 – Timepoint #1 | *I've worked in the field of HIV since the late 80s, so I'm not really against research and trying to advance in medicine. Put it that way.* |
|  | Helping HIV community | TASSO-8 – Timepoint #1 | *Like I said, help the community to create something that would make our lives easier.* |
|  | Logical next step | TASSO-9 – Timepoint #1 | *So this seemed like a pretty logical step since people have been doing that blood testing for sugar and all that, this would just make sense that they could do it [viral load testing] this way.* |
| Convenience and ease of joining device study as part of ATI trial | Already in ATI trial | TASSO-9 – Timepoint #1 | *I'm already doing the HIV study and it seems like an alternative to getting stuck with a big needle all the time. So I was like, "Why not?" I'm already doing one study, so what's the difference.* |
|  | Alternative way to draw blood | TASSO-4 – Timepoint #1 | *And I don't have to get a blood draw with a big, long needle like they normally do.* |
| Curiosity | | TASSO-10 – Timepoint #1 | *Just curious. It sounded interesting.* |
|  |  | TASSO-6 – Timepoint #1 | *Well, when [study coordinator] told me the premise of the gadget, and I saw how simple it was, I said, "Well, it can't hurt. So let's see where we can go with this... It just made me very curious.* |
|  |  | TASSO-13 – Timepoint #1 | *I was just curious about how would it be, or what was it about, how it works and stuff like that.* |
| Forward looking – viral load results from reliability component | | TASSO-1 – Timepoint #3 | *Yeah, I don't know what that is because I never got the results of what the lab came back with. So that, I don't know. But I'm just assuming that everything went well.* |
|  |  | TASSO-20 – Timepoint #1 | *I can see that it would be helpful. The only thing that's unclear to [me] personally as a patient receiving my viral load, I don't know who across the country is getting that information but as a patient I would like to have that information. So it's helpful in terms of like medical records for my own personal log.* |
|  |  | TASSO-12 – Timepoint #2 | *I don't know how the results compare to the actual blood draw… I don't know if the accuracy is there.* |
|  |  | TASSO-12 – Timepoint #3 | *It's just I've never been informed of any of the results… I'm not sure what's going on, on the technical end of it.* |
| **Experiences with Using the Device** | | | |
| **Reactions to Home-Based Blood Collection Device for Viral Load Testing** | | | |
| Positive reactions | Innovative; progressive | TASSO-10 – Timepoint #2 | *I think it's going to be the future, honestly, because people who have to check their blood sugar no longer have to go to a doctor, now they can just prick their finger. I think this is going to be the same situation.* |
|  |  | TASSO-10 – Timepoint #3 | *Just the prospect of the fact that it could be a new way to get blood drawn.* |
|  |  | TASSO-2 – Timepoint #1 | *When I think of innovation, and things that move it, that progressive, I don't see the downside. If the device is put together well, there's no real reservations.* |
|  |  | TASSO-18 – Timepoint #1 | *It seems like a lot of things medically are changing. So I expected something like this.* |
|  |  | TASSO-4 – Timepoint #1 | *I liked the advancement in the science and I said, "Well, how is that going to affect me actually getting information on my viral load?" Because I like to have my doctors go over my viral loads and my CD4 counts.* |
|  |  | TASSO-6 – Timepoint #1 | *When they first told me about its inception I was excited because I'm always excited for an advance in medicine. Your doctor's visit, it could be a horrible experience sometimes, being poked and prodded and just all these tests and things. If they can put a little teeny unit on the side of your arm that doesn't hurt, because it doesn't hurt at all, I've done it several times now, and then collect enough blood to actually tell most of the things they need to know about you, I'm all for it.* |
|  | Timely | TASSO-5 – Timepoint #1 | *Oh, it's about time, because it's self administer. After all these years, it's about time… Diabetics have been able to do it for so long. Right. So it just saves us one more trip to the doctor... It avoids that embarrassment, because now you can pick this little thing up and keep it moving.* |
|  | Convenient | TASSO-8 – Timepoint #1 | *It's nice. Like I said, it is less time. It helped me because in the future, I think I won't have to go to have the labs done, to go out of my house just to do it. So something nice.* |
|  |  | TASSO-8 – Timepoint #3 | *It's pretty convenient and I think it's pretty exciting to have this device right now, so whenever I need to get my viral loads in the future, hopefully I'll be able to use that instead of going to the clinic or to the hospital, being exposed to other people or wasting time going to the hospital.* |
|  |  | TASSO-10 – Timepoint #3 | *It's convenient. If I can do it at home, that's less hassle of going to a[n] office and having my blood drawn and all that.* |
|  |  | TASSO-7 – Timepoint #3 | *It's much nicer. That you don't have to leave out your house actually to get your lab work done. You can just stick the little thing on for five minutes. Sometimes it don't even take five minutes. Take it off and just stick it in the box, and there you go. So you don't have to go down to nobody's office and wait in line, then stick your arm out and take all those tubes for nothing. Or you got that little thing? I'm in. I'll buy. I'll invest.* |
|  |  | TASSO-11 – Timepoint #3 | *But with my device, I live close to a FedEx box, I walk four minutes and I'm done. That to me is, I want to say instant, but I'm going to call it extremely rapid… But if I could have that monthly to do my lab work, I would love to have it.* |
|  |  | TASSO-2 – Timepoint #3 | *I approve of the convenience that the Tasso device provides.* |
|  |  | TASSO-4 – Timepoint #1 | *And if the convenience is going to be awesome, because there's a lot of people who really don't like going to the doctor that often.* |
|  |  | TASSO-4 – Timepoint #3 | *But it's a great idea because a lot of times there are people who don't have transportation to their appointments to keep a proper monitor of their viral load.* |
|  |  | TASSO-20 – Timepoint #2 | *The entire process has been extremely convenient for me.* |
|  |  | TASSO-12 – Timepoint #3 | *Just the convenience of it. I could do it when I had time, if I wasn't tied down to an appointment, travel arrangements.* |
|  |  | TASSO-14 – Timepoint #1 | *Actually, it's a blessing... more of a blessing than a curse. A blessing because now I can get everything done at home and just ship it away without having to travel up to Philadelphia to get it done… But the convenience of home is I don't have to do nothing but just sit here, go across street to the mailbox, and drop it in. I'm done.* |
|  |  | TASSO-15 – Timepoint #3 | *It's kind of convenient, in my opinion, for someone that don't like to have to keep constantly going in just to get a blood draw or next test, all of that.* |
|  |  | TASSO-13 – Timepoint #1 | *Being as though you can just do it at home instead of going inside the hospital office and stuff.* |
|  | Convenient – time saving | TASSO-13 – Timepoint #3 | *The device is something good to use, and it save a lot of time.* |
|  | Easy to use; self-explanatory | TASSO-8 – Timepoint #1 | *It makes all the lab draws easy. I don't have to go eventually to work… I think it will be good for a whole lot because I won't have to go to the lab, get out of my house just to get the blood results or the blood test done.* |
|  |  | TASSO-10 – Timepoint #1 | *I guess I could do it at home is easier. It would save me a trip to get blood work. But that's yeah, I didn't because I wasn't really having a problem with going in for blood work, but it does make it easier for sure.* |
|  |  | TASSO-7 – Timepoint #2 | *It's self-explanatory, sir.* |
|  |  | TASSO-9 – Timepoint #1 | *It seemed simple to use. It's painless, it really didn't hurt. Does seem simple enough to use. You just slap it on. It seems to be a good idea. And with everything going on these days with COVID. I'm kind of upset I didn't think of it.* |
|  | Appreciation for device – coolness of device | TASSO-10 – Timepoint #2 | *The device is kind of cool-looking. It's got that little dial on it, it's kind of hip.* |
|  |  | TASSO-7 – Timepoint #1 | *My gut reaction when I first found out about it. I was like ooh its kind of cool that I don't have to go to that lab because I don't want to go to the lab so much.* |
|  |  | TASSO-13 – Timepoint #1 | *To me this is a pretty cool device… Yeah. It's pretty cool, man.* |
|  | Appreciation for device – concept | TASSO-20 – Timepoint #1 | *I was a bit impressed by the concept of being able to do home testing for viral load. I was excited about the idea, a little nervous about the pricking for the blood but everyone is…. I think it'd be helpful. I mean, so the test, the device in itself is a great invention. I think the idea of patients being able to take the device home, do their own viral load testing.* |
|  | Appreciation for device – features | TASSO-7 – Timepoint #3 | *I guess that it was compact and small, it wasn't big and heavy.* |
|  |  | TASSO-11 – Timepoint #3 | *The lightweight and the ease, and the comfort of it.* |
|  |  | TASSO-5 – Timepoint #3 | *I like the fact that it's small. It can fit in my drawer. Nobody has to even seen it if I have company coming over or whatever because it's a small little thing. It's in a small box. Just put it in a drawer, keep it aside. It's your private business anyway. It didn't have any special storage requirements.* |
|  | Appreciation – peace of mind | TASSO-11 – Timepoint #3 | *Personally, I love that it has made me feel extremely comfortable during the time I started the trial with the Tasso device... Even though I got blood work, that was just for me. And I didn't find out ‘til later that, that was a double insurance for me that I remained undetectable. It was fast, it was easy.* |
| Negative reactions | Needle aversion | TASSO-3 – Timepoint #1 | *I think some of the cons for me is, again, another needle, here we go.* |
|  |  | TASSO-4 – Timepoint #1 | *Yeah, I hate needles. Aw man, I got to stick myself now? Jesus.* |
|  | Initial hesitancy | TASSO-20 – Timepoint #1 | *Before actually using it, before it was fully explained to me, I was a little hesitant. But I love this idea of being able to test it at home. So I'm excited about it at this point.* |
|  | Initial confusion | TASSO-1 – Timepoint #3 | *It was a little confusing at first, cause I couldn't really understand it, but as time went on, it got better and it got easier, and I think I got used to it.* |
|  | Some device malfunctions | TASSO-5 – Timepoint #2 | *Well, I'm going to just put it nicely. It's cute. It looks nice, but it doesn't always fire, like a full collection. So you're sitting there holding it like, "Oh my goodness, did I do something wrong?" And then you realize, nope, it just was only going to fill up two slots.* |
|  |  | TASSO-13 – Timepoint #2 | *I think it's fine, but I believe some devices be faulty devices because a lot of times I'll use them and I put them on me and, I remember I put two on me at the same time and I got nothing out of neither device.* |
|  |  | TASSO-13 – Timepoint #3 | *I think they're good, but I think it's a lot of faulty devices because sometimes the ones that I take home and when I come in to do my visit and, and I put them on my arm, sometimes nothing comes up.* |
| **Experience with Testing Device: Perceived Ease of Use, Comfort, Safety and Pain** | | | |
| Perceived ease of use | Easy to use | TASSO-8 – Timepoint #1 | *It's easy. Super easy. It's not complicated.* |
|  |  | TASSO-10 – Timepoint #1 | *It was really a lot easier than I thought. I just popped it on and the blood came right out. It was packaged up and sent it… But it was one of the easiest things I've ever volunteered for, so… And the instructions were well laid out. It's step by step, so it was really simple to do.* |
|  |  | TASSO-10 – Timepoint #2 | *No. It's pretty simple to use. Put it on your arm and bag it up and send it off. I like it.* |
|  |  | TASSO-10 – Timepoint #3 | *It's pretty simple to use. Everything's prepaid, so I don't have to worry about any of that, either.* |
|  |  | TASSO-1 – Timepoint #1 | *But still in all, overall, it wasn't bad. It is easier than going to the doctor office. And they're sticking you. You got to go in there, you got to wait to be seen.* |
|  |  | TASSO-1 – Timepoint #2 | *It is easy. It's not complicated at all.* |
|  |  | TASSO-9 – Timepoint #1 | *It seems relatively simple.* |
|  |  | TASSO-3 – Timepoint #3 | *It's not a difficult thing to do. It's it's really not. Once you see it, it's done, they show you how to do it. It's not difficult to figure it out once you doing it on your own.* |
|  |  | TASSO-4 – Timepoint #2 | *100% ease of use.* |
|  | Easier than doctor’s office | TASSO-1 – Timepoint #2 | *I mean, I guess once we get down to it, it's a whole lot easier than actually going to the clinic and them sticking you with the needle and drawing the blood.* |
|  |  | TASSO-14 – Timepoint #2 | *The best part about it was a lot better than taking a needle at the hospital, so that was a perk for me.* |
|  | Read instructions carefully | TASSO-11 – Timepoint #1 | *Only thing that I thought was confusing and it was actually me, because I almost skipped a step because on a device that has that piece of tape that you tear off once you finish collecting so it can dry. Tape from the back and this tape is still there. So if you don't read the directions slowly, I think the directions are basically self-explanatory, but I guess it's when your first time when you go to do it, you're second guessing yourself, making sure that you read it correct.* |
|  | Special situations | TASSO-4 – Timepoint #1 | *The hesitation is because there are people who are just not coordinated enough.* |
| Use of device | Same way each time | TASSO-2 – Timepoint #1 | *So I think that it's necessary to follow directions, follow the method, and not make it up as you go along. So yes, I do it the same way, each and every time.* |
|  | Experimented with placements | TASSO-18 – Timepoint #3 | *I was telling [study member] that I try to vary the positions because I noticed early on when I would put the two devices side by side, you'd get a great result in one and you might not get anything in the other. I would start to place them not side by side, but one a little stair-step higher than the other one. I guess I experimented with the placements of them and I found that helped a little bit.* |
| Perceived comfort | Comfortable to use | TASSO-3 – Timepoint #2 | *I'm very comfortable with it. It doesn't bother me at all.* |
|  |  | TASSO-14 – Timepoint #2 | *Well, the way you all had it in the package made me very comfortable. You take it out the package, you can't pinch yourself with the needle. If you do, obviously you didn't read the directions anyway. So, as far as I'm concerned, the way it was set up is perfect.* |
|  | Comfort increased over time | TASSO-8 – Timepoint #2 | *It's getting easier because now I know how to do it by my heart. It is super easy. I memorized all the steps. I don't have to read anymore the paper that comes with it.* |
|  |  | TASSO-10 – Timepoint #2 | *Yeah, it was just easy. It was just getting to know the angles, because you've got to look in the mirror to see if the things are filling. Once you get that down, it just became habit now.* |
|  |  | TASSO-11 – Timepoint #2 | *But with repetition, comfortability comes.* |
|  |  | TASSO-2 – Timepoint #2 | *I think anything done with repetition should become easier.* |
|  |  | TASSO-2 – Timepoint #3 | *I think over time you get more comfortable, at least for myself, first and second. The first three times you feel kind of nervous. Am I doing this right? I don't want to miss a step.* |
|  |  | TASSO-18 – Timepoint #3 | *I've been doing it for a period of time now that I feel more and more competent… the more you do it, the more you build your confidence with it and feel good about it.* |
|  |  | TASSO-5 – Timepoint #2 | *Well, of course, the more you do something, the easier it becomes.* |
|  |  | TASSO-5 – Timepoint #3 | *Comfort increased over time.* |
|  |  | TASSO-12 – Timepoint #2 | *It was always high, but the more you do it, the less strange it seems.* |
| Perceived safety | Safety reason – no blood splatter | TASSO-3 – Timepoint #3 | *You don't have no blood splatter. So yeah, I think it's very safe.* |
|  | Safety reason – no large needle | TASSO-8 – Timepoint #2 | *Because there are no big needles. It is not a sharp.* |
|  |  | TASSO-8 – Timepoint #3 | *I don't see how it couldn't be safe, because you don't have access to any pointy part. There's no needle that we have access to. Once you put in your skin, just press the button and the needle goes back in right away, and yeah, it's pretty safe.* |
|  |  | TASSO-6 – Timepoint #2 | *I think it's safe, because for one thing, I know people, I know they don't like needles. Okay, that's fine. It's not a great big giant needle.* |
|  | Safety reason – limited room for contamination | TASSO-7 – Timepoint #1 | *Oh yeah, its safe cause it's fresh out the pack. Everything is sealed and you unseal it yourself, so yeah.* |
|  |  | TASSO-2 – Timepoint #2 | *The reason I say that is because the device has little room, actually little or no room, for contamination. And contamination with blood draw, for me, is the most important thing.* |
|  | Safety reason – alcohol swab provided and no risk of infection | TASSO-10 – Timepoint #1 | *Yeah, I would say as long as you use the alcohol swabs, there's no chance of infection. I don't see a problem with it.* |
|  |  | TASSO-10 – Timepoint #2 | *I think it's safe. As long as you clean the arm and put it on. It's all sealed when you get it so it's just a matter of cleaning the arm, the spot it's going on, with alcohol. I don't see how you could get any kind of infection or contamination as long as the area is clean.* |
|  |  | TASSO-10 – Timepoint #3 | *Yeah, as long as you clean the area before you use it, I don't see any problem. I didn't run into any problems, nothing infections or anything like that.* |
|  |  | TASSO-2 – Timepoint #3 | *Yeah, I really do, because the safety is so... I think the sanitation portion is covered because we cleanse our skin with the alcohol pad, and then because it's so close on you, there's no air contamination or other outer contaminations that can get in because of the sticky tape. That's the one that keeps it pressed up against the hole that's being made. I think it's self-contained safety is, I guess, the best description I can give it.* |
|  |  | TASSO-3 – Timepoint #1 | *The device is already sanitized because it has the cap on it, it has the little strip on before it touches your arm. It comes with the alcohol pad so you make sure the area is clean before you put it on. I feel like it's really safe.* |
|  | Safety reason – need to use immediately upon opening package | TASSO-2 – Timepoint #1 | *When you take it out the package, use it immediately. Some people get distracted. For instance, I can imagine, this is just hypothetical. If someone was to get distracted, pulling off all the plastic, taking it out of the wrapper, and it's just sitting out in the open, then it's not safe. But if you use it immediately as directed, I see it being safe and convenient.* |
|  | Safety reason – no risk of COVID-19 | TASSO-14 – Timepoint #1 | *COVID-19... Sometimes we may forget to wash our hands, or we forgot to put gloves on, or you don't know if someone got something that they don't even know they have. By me doing it at home, in the comfort of my home... Get the blood work, put it in a container like you're opposed to, ship it off. I know where my hands been. I know where my skin been. I know what I did. So it's kind of like a comfort level.* |
| Perceived pain | No pain or limited pain | TASSO-9 – Timepoint #3 | *No. I have a high tolerance for pain, so it doesn't really bother me. It's just like a little stick. Well, at least that's what I think because I have tattoos all over. Tattoo artists love me. I just lay there and zone out.* |
|  |  | TASSO-18 – Timepoint #3 | *Well, I haven't had any adverse effects. The needle prick is not painful. It's not intrusive… Yes, I feel safe with it.* |
|  | No hazard for self-harm | TASSO-20 – Timepoint #2 | *Outside of the intentional pricking of my skin, I don't find it as a hazard to hurting myself.* |
|  | Not invasive | TASSO-7 – Timepoint #3 | *It's not invasive. Yeah, and it's not painful.* |
|  | Less painful than regular needles | TASSO-8 – Timepoint #1 | *It doesn't hurt like regular needles… You can barely feel the pain. I thought it would be hurtful just like when you get tattoos, but no, it's... you barely feel the needle.* |
|  |  | TASSO-8 – Timepoint #3 | *It's not painful like regular needles.* |
|  |  | TASSO-10 – Timepoint #3 | *I mean, it's less painful than someone putting a needle in your arm.* |
|  |  | TASSO-4 – Timepoint #1 | *Nowhere near as painful as getting the full needle blood draw. Way less... It's less painful than the normal blood draw.* |
|  | Less painful than finger stick | TASSO-11 – Timepoint #3 | *A finger stick, it's less painful than a finger stick drawing.* |
|  |  | TASSO-4 – Timepoint #2 | *Yes, for me it's less than a finger prick. Because in a finger prick it leaves this numbness on the tips of your fingers, so once you do the little thingy in about an hour there's no pain.* |
|  |  | TASSO-20 – Timepoint #2 | *Better than a finger prick. Like for finger check, like if you're checking your sugar for diabetes… where you prick your finger, that stings or burns and to some extent after the prick you still feel it. I feel the pinch. It's a very light pinch when I'm doing the test, but once I release it, there's no more pain. I don't feel it. It doesn't bother me.* |
|  | Sensation – little punch | TASSO-8 – Timepoint #2 | *It doesn't hurt. It is just a little punch and that is it. It doesn't hurt.* |
|  | Sensation – pinch | TASSO-8 – Timepoint #3 | *It's like a little pinch, so almost nothing.* |
|  |  | TASSO-10 – Timepoint #1 | *I noticed a little pinch, but no more than like a needle would.* |
|  |  | TASSO-7 – Timepoint #1 | *It's nothing. A quick little pinch.* |
|  |  | TASSO-1 – Timepoint #1 | *It's a little pinch when you first put it on, you get that little stick. That initial stick, but then that's it… It's like a little pinch, a little stick. And before you have any time to flinch, it's over with. You put it on and you push the red button it goes then and that's it.* |
|  |  | TASSO-3 – Timepoint #2 | *A pinch. Like somebody pinched you or like if I had taken a needle, like a safety pin. I'm going to use that, and just stuck myself with it. It's just that simple.* |
|  |  | TASSO-5 – Timepoint #2 | *It's a pinch.* |
|  |  | TASSO-20 – Timepoint #1 | *A pinch… I compare it to a pinch.* |
|  |  | TASSO-15 – Timepoint #2 | *Just like a pinch. That's all.* |
|  | Sensation – easy pinprick | TASSO-18 – Timepoint #3 | *It's just an easy pinprick. It doesn't go deep. It isn't painful. For years, I'd go to [Name of Laboratory] to have blood drawn, two weeks before my medical visit to the doctor. You go there and the phlebotomist has to hit the vein and that sometimes can be painful and that sometimes can leave a scar depending on how they do it. Some phlebotomists are very gentle and some of them are very rough. Again, I've been doing this for 21 years. With the pinprick that the Tasso does, that doesn't leave a scar and it's not actually pricking the vein like they do at the labs. I feel more comfortable with it than having to hit the vein.* |
|  | Residual marks left after punctures | TASSO-3 – Timepoint #3 | *I think my only issue is where I was sticking myself at on my arms and like the marks... It does leave little pin marks, which is understandable because I am sticking myself with a needle stuff. But it's no discomfort again, like I said. Like it doesn't hurt, it's not painful at all.* |
|  |  | TASSO-4 – Timepoint #3 | *You mean issues like leaving a spot on my skin because I usually tend to hit almost the same area, so there's like a dark little spot on my skin because I actually got the same spot two or three times.* |
|  |  | TASSO-6 – Timepoint #2 | *And it's not that it's a big thing, but at the same time, it was kind of disappointing. And after you done done it and done it and done it, after these four or five times I've done it, I've got these puncture marks in my arm. And you can feel them. By the time that one heals, you're doing it again. Then by the time that one heals, you're doing it again. So when you go to run your hand down your arm, it feels like mosquito bites.* |
|  |  | TASSO-20 – Timepoint #3 | *The only concern that I would have are the dark spots on my shoulders from all of the uses.* |
|  |  | TASSO-15 – Timepoint #2 | *The spot from the needle being stabbed. It's different from when you get to a blood draw. I don't have any spots from getting a blood draw, but only from using the machine, I'll get little spots from being stabbed.* |
| **Factors that Would Promote or Prompt Using Device** | | | |
| User support | Demonstration in clinic and coaching from study nurses | TASSO-1 – Timepoint #1 | *Now if he [study coordinator] wouldn't have did it in the office with me, maybe my expectation of it would have been completely different, but because we watched the video and then he did it in the office and told me everything I needed to know then. So that's why it was so easy for it.* |
|  |  | TASSO-9 – Timepoint #2 | *I would say more of [study coordinator]'s demonstration. I'm more hands-on so I'm more of a kinesthetic person. I have to learn by doing it myself but watching [study coordinator] actually do it and watching him do it on me, I learned better. But then just trying to do it on myself from memory, it's how I learn.* |
|  |  | TASSO-18 – Timepoint #3 | *I believe [staff member] was really the most significant part of all of that. He was pivotal in making me feel comfortable, taking away all of my fears or awkwardness and making it all very comfortable and safe and easy to process.* |
|  |  | TASSO-4 – Timepoint #2 | *The demonstration in the lab.* |
|  |  | TASSO-5 – Timepoint #1 | *And then the self demonstration, and showing me how to do it, and making sure I was comfortable with it, that made it so much easier and got my questions answered.* |
|  |  | TASSO-6 – Timepoint #3 | *Well, I did watch the video, but that didn't do it for me. It was actually our study assistants that physically "Do this, do that" right in front of my eyes. "Do this, do that" and made me repeat it a couple of times before I actually did it... or before they did it, they showed me what they're getting ready to...for the first couple times they did it. Once I saw them do it, I could remember the steps... And it only took the one-time for them to show me.* |
|  |  | TASSO-15 – Timepoint #2 | *For me, personally, it was the one-on-one hands on at the clinic.* |
|  | Video | TASSO-8 – Timepoint #2 | *The video. I am more of a visual person, so the video helped better than the other ones.* |
|  |  | TASSO-11 – Timepoint #2 | *So the video… I didn't want to read everything.* |
|  |  | TASSO-2 – Timepoint #2 | *The video.* |
|  |  | TASSO-14 – Timepoint #1 | *I watched the video on my tablet. I was like, "Oh, that makes sense. Okay, I can do it that way."* |
|  | Instructions | TASSO-10 – Timepoint #3 | *Obviously it has the step-by-step instructions so it's kinda like an IKEA instruction. Yeah, they're pretty comprehensive. It's right there step-by-step.* |
|  | Combination of above | TASSO-3 – Timepoint #2 | *It was a combination of. Because when they first were showing me about it, they showed me the video, I watched how it was done, then the nurse in the clinic, she kind of demonstrated and that made it even more visual for me. But when I read the directions, it was just real clear and simple. It was just real simple. The directions, they're so simple... It's not just the words. It kind of gives you a play by play on how to do it. So that's what made it more simple for me. I'm a visual person. I like to be able to see stuff. Show it to me and I can get it.* |
|  |  | TASSO-20 – Timepoint #2 | *So I'm a visual and hands on type person so the video helped a lot. The instructions are simple but I think the demonstration inside the clinic also helped. It took away the initial fear of pricking myself. So she did one and then I did one which made me comfortable with it.* |
| Practice or trial and error | | TASSO-9 – Timepoint #2 | *Because that's the only way I'll learn, is if I'm doing it and you just tell me, "Okay. Hit that button combination," and then that's how I learn.* |
|  |  | TASSO-18 – Timepoint #2 | *I've learned again from trial and error to not put them side by side. I don't know if that was effective or anything, but instead of putting them on at the same exact time, sticking them in like side by side, around the exact time, I now do one a little higher and one a little lower. Like I said, stair-stepping them and a few minutes apart. For some reason, it looks like it's not pulling from the one place.* |
| Blood flow strategies – heat | | TASSO-2 – Timepoint #3 | *I have been given the little hand warmers. I think with just vigorously rubbing my arm with a warm rag could do the same thing, and that's what I've opted to do, as opposed to using the hand warmers kind of thing, because it slips, and sometimes it doesn't stay in place as you're trying to get the friction on the arm with the warmth of the hand warmer. I think a warm rag and some arm curls has been better getting blood circulation into the arm than just warming your arm.* |
|  |  | TASSO-15 – Timepoint #2 | *But it works best as soon as you take a shower or you put a hot rag on out the shower or like a hot rag and you get a better reading. You can get more of a blood draw that way, than just rubbing it and making it warm. That's in my opinion.* |
| Blood flow strategies – heat | Coming out of shower | TASSO-7 – Timepoint #2 | *It works so much better when you get out the shower.* |
|  |  | TASSO-11 – Timepoint #3 | *I find it best if I either do it fresh out of a nice hot shower.* |
|  |  | TASSO-5 – Timepoint #2 | *After I take a warm shower.* |
|  | Heating pad | TASSO-12 – Timepoint #2 | *Yes, with a heating pad.* |
|  |  | TASSO-14 – Timepoint #1 | *So when I did it that way, you all gave me the little heating pad to warm it up a little bit quicker, but like the lady said in the video, if you rub your hand good enough, you could feel the warmth coming through... And believe it or not, with that plus the heating pad just because it was there, it did a damn good job.* |
|  | Heating blanket | TASSO-11 – Timepoint #1 | *I saw the nurse put a heating blanket because I'm a hard stick, she put a heating blanket on my arm to get the blood flow and that's what I was told, you're getting the blood flow and warming up your veins.* |
|  | Warming pack | TASSO-13 – Timepoint #2 | *Well in the box, there's like a warming pack thing and then you take that and you rub it against your arm. Say if your arm is cold, you will use that. But if your arm is already warm then you might not need it.* |
|  | Air Dryer | TASSO-11 – Timepoint #1 | *I used my hairdryer.* |
| Blood flow strategies – massaging or rubbing | | TASSO-8 – Timepoint #3 | *I did rub my skin a little bit hard, and that's it. It works pretty well.* |
|  |  | TASSO-10 – Timepoint #2 | *A hand massager I use, which works great. It gets the blood flowing really good.* |
|  |  | TASSO-10 – Timepoint #3 | *I used a massager… But I used an electric massager, which cut the time down. I think you get like six minutes, but I usually got my blood out within two minutes by using the massager. It brought enough blood up into the arm.* |
|  |  | TASSO-1 – Timepoint #3 | *Yeah, just the warmer and massage it a little bit, rub it. When I didn't have the little warmer, just rub it real good, and massage it a little bit, and that was it. Makes it seem easier.* |
|  |  | TASSO-3 – Timepoint #3 | *Like massage my arm. I would massage my arm a little bit. And when I put the device, once I stick the device on, I would like kind of massage it around the area where the needle has gone into my arm and it helps the flow, keep the blood flowing and it works.* |
|  |  | TASSO-11 – Timepoint #1 | *The only part I don't like, when you have to rub vigorously on your arm. So I did it a little, I'm not going to lie.* |
| Blood flow strategies – moving or shaking arm | | TASSO-1 – Timepoint #2 | *I give it a little help. I might just sling it down, shake it maybe once or twice and everything will just flow right on down.* |
|  |  | TASSO-5 – Timepoint #2 | *Yes. I was flexing the back of my arm, anyway, so it worked.* |
| Other – mirror | | TASSO-6 – Timepoint #1 | *I will say this much, putting it in the side of your arm is a little awkward unless you're looking in a mirror. If you're not in a situation where you're looking in a mirror, it can be a little awkward putting it in your arm because once you face it away from you, you can't see the blood collecting. It's on the side of your arm. To be honest, with the positioning of where I have to put it on my arm to actually get it to work, it's high up on the shoulder, which means I definitely can't get around that to see it unless I'm in the mirror to see the actual chamber is filling or the slots filling.* |
| Other – hydration | | TASSO-11 – Timepoint #2 | *I guess if you're hydrated, I think that should be in there too. Because you know, when you're drawing blood if you aren't hydrated you have a hard time.* |
| Other – no lotion | | TASSO-11 – Timepoint #3 | *Make sure you don't have any lotion, I found that out, I'm a baby oil guy. You can't have any of that on, because it's definitely not going to stick.* |
| **Difficulties with Using the Device** | | | |
| Devices not filling up completely | | TASSO-8 – Timepoint #3 | *Probably two or three times, it didn't fill up. I don't know if the needle was broken inside, even though I felt a punch on my skin. But no blood came out, I don't know exactly why.* |
|  |  | TASSO-10 – Timepoint #2 | *Oddly enough today it didn't, it only got three and two covered, but normally I get all four covered so I might have just put it in a spot that wasn't as strong as previous. But it fills up enough I guess for them to get the blood work.* |
|  |  | TASSO-10 – Timepoint #3 | *We just had a couple along of the however many we did that were having trouble drawing blood, but it could have been the device. It could have just been my circulation. So, we only had maybe two times we had trouble with one of the devices, but the other device got enough blood.* |
|  |  | TASSO-11 – Timepoint #2 | *I only filled a half a chamber in one device. But there's blood. You can see there's blood in there at the top... So I know I know what I'm doing. But if it doesn't work, that bothers me.* |
|  |  | TASSO-1 – Timepoint #2 | *For some strange reason, when I did the one I think I got maybe two full dots on that one. But then the second one that I put on there, for some strange reason when I put it on, I went to hit the button to mash the injection in, that little needle in, for some reason it didn't go in and I got nothing out of that one.* |
|  |  | TASSO-2 – Timepoint #2 | *We talked about the partial fillers, that's a possible defect. It doesn't mean that, it means that I need to do it eight minutes maybe, so it might just be running slow.* |
|  |  | TASSO-9 – Timepoint #2 | *And there's been a few times where one fills up and the other one doesn't.* |
|  |  | TASSO-9 – Timepoint #3 | *There are still some issues with some of them not filling up completely.* |
|  |  | TASSO-18 – Timepoint #3 | *I try to get the device filled, but sometimes it just won't fill completely. I feel like maybe I've done something wrong, but I don't think so.* |
|  |  | TASSO-3 – Timepoint #1 | *A couple of them, like the one yesterday was the very first one that I used, the blood didn't flow into one of them. Yesterday at the doctor's office it seemed like it was clogged right as it gets to the point where it starts to flow into the little dock. It was just like it was sitting there like it was clogged or something. I don't know if it was the device, if it was blood, I don't know what it was, but it didn't flow through like it normally would. That's the only thing that's like okay, is it something I'm doing wrong? Is it the device itself?* |
|  |  | TASSO-3 – Timepoint #2 | *So when I took it off of my arm, you can see where the blood is sitting in the little thing on the back. You could see it sitting there, but it wasn't going through. So I don't know if it had something to do with my arm.* |
|  |  | TASSO-4 – Timepoint #2 | *Just the not filling up.* |
|  |  | TASSO-5 – Timepoint #2 | *And it does take a long time to fill up.* |
|  |  | TASSO-12 – Timepoint #2 | *Even in the clinic when the nurse and the doctor put it on, both of them don't fill up. I'm experiencing the same thing at home where one might, but the other one won't. I don't think I've had both of them fill completely up.* |
|  |  | TASSO-12 – Timepoint #3 | *My only observation is that sometimes the device would not fill up completely… my only concern with it is doing, using the device making sure that there's enough sample to analyze.* |
|  |  | TASSO-14 – Timepoint #2 | *I just couldn't fill it up all the way… The first one filled up with no issue whatsoever. The second one, I think it got to the second dot, going into the second dot, and I just couldn't get nothing out of it with the time allotted.* |
|  |  | TASSO-13 – Timepoint #2 | *The device is fine. It's just that one problem, which it's not drawing the blood. The blood is not gone through the machine like it's supposed to. So to me, there's something wrong with the device.* |
| Devices not filling up completely  Blood coagulates too soon | | TASSO-2 – Timepoint #3 | *Sometimes, let's say for me, when I open the envelope to take out the boxes, I think the easiest thing to do is to put the time and date, because there's been time where I closed the box without labeling. That little box, the section to add the date of collection and time of collection, it was written on top of the box, it was collected at such-and-such a time on such-and-such a date because I had already sealed the sticky portion of the box.* |
|  |  | TASSO-4 – Timepoint #1 | *Not getting enough in the collection… The blood coagulates pretty... certain people's blood is going to coagulate pretty quickly in that chamber. One time I pulled it off and there was a big gelatin glob of blood still on my arm.* |
| Device not sticking on arm enough | | TASSO-7 – Timepoint #3 | *One time it had fell off after like 10 seconds.* |
|  |  | TASSO-11 – Timepoint #3 | *Sometimes the tape that sticks to your skin, sometime that may not work.* |
|  |  | TASSO-9 – Timepoint #2 | *The only other difficulty I had was when the one wouldn't stick to my arm.* |
|  |  | TASSO-9 – Timepoint #3 | *I had a few devices fall off my arm after a minute or two.* |
|  |  | TASSO-5 – Timepoint #3 | *I just want the glue to be stickier or stick better. I'm always afraid it's going to fall off. It just doesn't feel like it's holding tight. But other than that, no. That's the only thing. Just give us a little more glue or a little more stickiness on the tab part that sticks to the arm.* |
| Device not sticking on arm enough | Unable to reuse device | TASSO-9 – Timepoint #2 | *I only had the one where the adhesive didn't stick to the arm. So that stayed on for a minute and then it fell off. I don't know what happened there, but I couldn't get it back on because it fell face down, the adhesive side down on the floor so I just didn't even bother trying to put it back on.* |
|  |  | TASSO-5 – Timepoint #2 | *One time when I moved and it popped off, I couldn't get it to stick back on.* |
|  |  | TASSO-6 – Timepoint #2 | *It fell off… And it kind of scared me, because when it fell off, the sticky part hit the floor… So I can't put it back on my arm now cause it's not sterile. And now I'm sitting here with alcohol pads, trying to clean little cat hairs and things off, because it literally stuck to my carpet. I'm like, "Well, I'll be damned." But that only happened once. Other than that, they always stick.* |
| Minor blood spillage | | TASSO-18 – Timepoint #1 | *The first time I used it at home… We had a little spillage.* |
|  |  | TASSO-18 – Timepoint #2 | *One time I put it in and it just fell right out. So once you press it down and hold it for those three to five seconds, that the needle is going in. Well, I did that. And then I went and turned and did some other thing and it just fell right out of my arm and fell to the kitchen floor. And then there was some spillage.* |
| Other difficulties or issues | Mysterious second sound after initial push | TASSO-20 – Timepoint #2 | *There was one concern that I had with it, and it still does what it needs to do, but I've noticed that when I'm, according to the instructions, it tells me to put the device in my arm or wherever I'm going to draw the blood from, push the button in for two seconds, and release. And it releases but I noticed that when I remove the device from my arm, you hear it click as if the needle is retracting a little bit more. And I'm wondering if that's affecting how fast or slow the device can collect a blood sample. Yeah. It just made me wonder if that second pop was the needle retracting or what because it sounds like it is. It even feels like it.* |
|  | Inconvenience of having to wear band-air after use | TASSO-5 – Timepoint #3 | *I was going to say the band-aid, but that's not a problem. I just hate having to wear it. Because it's right on the back of your arm and you're on your way to the store and you're like, "I don't need that to fall off," but that's all.* |
|  | Irritation from band-aid | TASSO-3 – Timepoint #2 | *The only issue that I've been having, and I think it's me personally, my skin, like the band-aids, seemed like I have an irritation on my arm where I'm putting the band-aids at.* |
|  | Remembering to write date and time of blood collection | TASSO-2 – Timepoint #3 | *Sometimes, let's say for me, when I open the envelope to take out the boxes, I think the easiest thing to do is to put the time and date, because there's been time where I closed the box without labeling. That little box, the section to add the date of collection and time of collection, it was written on top of the box, it was collected at such-and-such a time on such-and-such a date because I had already sealed the sticky portion of the box.* |
| **Perceived Advantages of the Device** | | | |
| Convenience | | TASSO-2 – Timepoint #1 | *That whole convenience piece. Not just for people who are able, but for both people who might be challenged in various ways.* |
|  |  | TASSO-2 – Timepoint #2 | *So I think that cutting edge part and how convenient and expeditious it is.* |
|  |  | TASSO-4 – Timepoint #2 | *Convenience, convenience, convenience.* |
|  |  | TASSO-4 – Timepoint #3 | *But even with that little thing there, it's still 100% worth it for HIV patients. It adds a convenience to care that not a lot of people are going to think about, but when it's explained to them they'll understand.* |
|  |  | TASSO-5 – Timepoint #3 | *I like the convenience of the device.* |
|  |  | TASSO-20 – Timepoint #2 | *I think just the convenience of being able to do... I'm excited about the idea of being able to do this at home, so I think that's one of the things that I like most.* |
| Convenience | Time (and cost) saving | TASSO-10 – Timepoint #2 | *It saves cost, it saves time. It's going to be a good move.* |
|  | Reduces need for doctor or clinic visits | TASSO-8 – Timepoint #1 | *Like I said it would make my life easier. If I eventually don't have to go to the hospital or to get my labs done. That's exciting, to make things easier for me, for everyone.* |
|  |  | TASSO-10 – Timepoint #2 | *Like I said, I don't know if I like these devices as much as I like the idea that if it one day eliminates having to go to the doctor for blood work...* |
|  |  | TASSO-7 – Timepoint #1 | *This saves me from going to the lab, getting up, coming out my house, going there, waiting in line, getting pricked, making my arm hurt. This one is way better and its faster.* |
|  |  | TASSO-12 – Timepoint #2 | *Well, if it's something that could negate the necessity to go to the doctor's office and actually have a blood draw done, I think it's great.* |
|  | Long-term benefit of not having to go to doctor or clinic | TASSO-1 – Timepoint #2 | *I'm looking long-term. Maybe there's some day and that would be where I wouldn't have to go to the clinic. We could just do it this way and mail it in, and that would save a whole lot of time. Overall, I'm looking long-term.* |
|  |  | TASSO-18 – Timepoint #3 | *I can see in the future that this will benefit people who won't have to go to their doctor's office so much. I know that's changing gradually for a lot of people. Once upon a time I had to see my doctor every three months now I only have to see them every six months.* |
| Helpful during COVID-19 pandemic (e.g., telehealth) | | TASSO-2 – Timepoint #2 | *In the middle of this [COVID-19] pandemic, with people not being able to move, come and go freely, that this could be a quicker method to get information to your doctor, since we've transitioned into… Zoom calls and telemedicine, that whole revolutions that's taking place right now.* |
|  |  | TASSO-14 – Timepoint #1 | *No, because that's what we have to do with my job for the COVID testing. I do it every two weeks. I'm one of the people that volunteered. I take the saliva, put it on a Q-tip, break it, put it in a tube, go to the FedEx office and drop it off. So, no, I actually prefer it that way because I know it's getting done.* |
| Control over healthcare; empowering | | TASSO-18 – Timepoint #1 | *It's empowering. 21 years of living with this [HIV], it's been quite a ride, quite the journey. You go from thinking you're going to die to learning you're never going to die to then living with it and then accepting so many things. It's such a journey of knowledge and understanding your body. This is empowering because I am taking such a front role in my own care… It's giving the power to me to be a part of my own health care.* |
|  |  | TASSO-6 – Timepoint #2 | *I can do this myself. I don't have to have a degreed doctor put this on my arm. I don't have to worry about long needles, then maybe I hit a nerve or whatever have you. I think it is a great thing towards helping us be more self-motivated in self-care.* |
| Confidentiality and privacy | | TASSO-8 – Timepoint #3 | *I guess the privacy.* |
|  |  | TASSO-5 – Timepoint #1 | *For the device we're discussing, the confidentiality factor has always been very, very important to me. So for me, it immediately went to confidentiality… That stigma for my generation still exists… It's the future of monitoring.* |
| Fast way to get medical information | | TASSO-2 – Timepoint #2 | *So collecting a blood specimen at night at home and sending it out the same night could be life saving, so it really has a completely different appeal, simply because it gets the medical information, from inside the patient, in the doctor's hand faster.* |
|  |  | TASSO-4 – Timepoint #3 | *I think it helps the clinics get your viral loads a lot sooner and makes it convenient enough that they can take your viral load, a major viral load test at the clinic, and then in between visits they can also help monitor your viral load by the patient sending them in on a more timely fashion type thing. So, instead of having to have an appointment to say, "Hey, we need you to come back in a month so we can redo your viral load," say, "We're sending this kit with you. We'll send you a reminder of when you should do this kit, send it in, that way we can check your viral load and we don't have to have you run in in between your normal visits." For me, being on this study, they were having me visit every week. So, every week I have to take the time out to come back to the office.* |
| Obviates need for venipuncture | | TASSO-8 – Timepoint #2 | *I don't have to be looking for any veins or anything like that.* |
| Obviates need for large needles | | TASSO-4 – Timepoint #1 | *Because now I won't have to take the big needle. I won't have to take the big needle now.* |
| Handheld device | | TASSO-11 – Timepoint #2 | *I love it that it's handheld.* |
| Device kit | | TASSO-2 – Timepoint #1 | *It's prefrabricated so well… You could tell it's a lot of thought that went into delivering and bringing this product.* |
|  |  | TASSO-14 – Timepoint #2 | *Actually the whole convenient package.* |
| Device will improve | | TASSO-6 – Timepoint #2 | *I am kind of excited because if they do the tweaking like they should, this could be a great opportunity to give the average person to come up on their own personal efficacy.* |
| Benefit to society and to self | | TASSO-1 – Timepoint #2 | *I'm always excited about something new that's coming out that's going to benefit somebody else. But more important I get the benefits from this, as well.* |
| **Perceived Concerns about the Device** | | | |
| Limited concerns expressed | | TASSO-7 – Timepoint #1 | *No, not at this moment, I don't.* |
|  |  | TASSO-11 – Timepoint #3 | *There's nothing to be afraid on that device.* |
|  |  | TASSO-1 – Timepoint #1 | *No, no real concerns. No.* |
|  |  | TASSO-2 – Timepoint #1 | *Me personally, I can't imagine being squeamish about it. And the fact that the blood doesn't get all over the place, I don't see it being an issue for other people who very well may have some apprehensions like that. I think more than anything else, it is so compact. The blood delivery is so... It's minute. It's a really small amount of blood once you pull the device off your arm. And then the little incision mark that it makes is again, is convenient. It has no problems to it right now. No contamination risk, just sticking the arm is easy to care for, so it's not a lot of blood leaking from your arm. It really doesn't present anxiety and apprehension.* |
|  |  | TASSO-6 – Timepoint #2 | *No, not at all.* |
|  |  | TASSO-15 – Timepoint #1 | *No, not at this moment, I don't.* |
| Limited concerns | No blood aversion | TASSO-9 – Timepoint #1 | *No, not really. Like I said, it's a shorter needle, so I really don't have any concerns about that blood. Blood averse? No… Nothing really phases me blood-wise.* |
| Need for transportation to mail blood | | TASSO-9 – Timepoint #2 | *If they're elderly and there's transportation issues, they can get the devices FedEx to them, do it at home and then just have FedEx pick it up at their door.* |
| Developing dependence to device | | TASSO-12 – Timepoint #1 | *What if we're going to be dependent on it from our blood work?* |
| Possible contraindications | | TASSO-2 – Timepoint #2 | *Unless somebody has some kind of shakes problem, from some kind of medication side effect, if they are not stable-handed, then they need help... Because there are, especially if you think about elderly people using it, or someone convalescing in a nursing home, they might need assistance.* |
| **Suggestions to Improve the Device** | | | |
| Device mechanism or placement | Improved suction | TASSO-11 – Timepoint #2 | *I think if you put the suction on it a little stronger.* |
|  |  | TASSO-11 – Timepoint #3 | *And the other thing, if maybe they could work on, is the suction or the withdrawal. Because sometimes it makes you feel bad if you only get one circle of blood, when you know there's four chambers to fill, or you get none, but you can see it inside the chamber.* |
|  |  | TASSO-2 – Timepoint #1 | *A stronger vat or suction that makes that flow without any manipulation. Does that make sense?* |
|  |  | TASSO-4 – Timepoint #2 | *Honestly, the only thing I say is that little suction piece because you actually can hear it when the lancet goes back it makes a little almost like a kissy noise. If it had more of a way of letting you know, "Okay, we're working." Rather than trying to see it in a mirror, if it had a something like more audible click that says, "Okay, samples are happening." But it's not necessary, it's just my little quirk about it.* |
|  |  | TASSO-6 – Timepoint #1 | *It's gravity and the device. I don't know if maybe they need to tweak the needle maybe, perhaps, or the suction of the collection device tube, or is there something maybe you could put in the tube that doesn't.* |
|  |  | TASSO-12 – Timepoint #2 | *Well, I don't know how that can be done. I'm not sure what the principles are behind it. I don't know if there's a vacuum in there or something or, if the puncture is supposed to be large enough to make me bleed that much.* |
|  |  | TASSO-15 – Timepoint #3 | *I think it's the needle may not be long enough where he would get more of a blood draw or anything like that.* |
|  | Way to prevent clogging or coagulation | TASSO-11 – Timepoint #3 | *And the other thing, if maybe they could work on, is the suction or the withdrawal. Because sometimes it makes you feel bad if you only get one circle of blood, when you know there's four chambers to fill, or you get none, but you can see it inside the chamber.* |
|  |  | TASSO-1 – Timepoint #2 | *If there's some kind of way I guess where it wouldn't get clogged up.* |
|  | Find different placement | TASSO-15 – Timepoint #3 | *Maybe. I guess it depends. I was telling them that maybe they need to try other parts of the body that may help to get a better reading instead of just on the shoulder, the upper shoulder, that's where I was doing it.* |
| Device features | Smaller (more compact) or narrower device | TASSO-10 – Timepoint #1 | *Yeah, it would be nice if it was a little bit smaller.* |
|  |  | TASSO-9 – Timepoint #2 | *Maybe make the round part a little narrower… Because not everyone has... My bicep area isn't very big and I do notice sometimes when they're next to each other, sometimes they might hit, so I always have to place the one a little bit lower than the other one. But it just seems like when they're next to each other they're knocking sometimes, for people that have narrow arms like me.* |
|  |  | TASSO-3 – Timepoint #1 | *But maybe a smaller type thing.* |
|  |  | TASSO-3 – Timepoint #2 | *So if it was just where you just stuck it on your arm and it flows right into the device, right into the thing, the circle part of it, I don't know. Maybe it would flow better. I don't know. I'm not a technologist. I don't know how they got this device to operate itself, but I guess it would be a little bit more compact.* |
|  |  | TASSO-4 – Timepoint #2 | *And it's so easy to use already, but they might be able to make it a little smaller. That would make it a little bit more convenient with a little smaller maybe, something like that.* |
|  |  | TASSO-3 – Timepoint #3 | *That would be my only thing is the device being smaller because I think... And I think that if it was smaller, maybe it would be, I guess... Say if I was out and I needed to do it, it wouldn't be so noticeable that I was putting the device on me. So that's the only thing I feel it could change is like the size of it. Because people might be out and instead of having that big bulky device, something smaller, I could just sit there do it and nobody would really take a notice to it. But it is not a concern because for the most part I do it at home.* |
|  |  | TASSO-20 – Timepoint #1 | *It's big and bulky. In terms of design, I don't know what goes into the design work and I'm not critiquing anyone's work because it is actually amazing but I think being big and bulky is maybe a thing if the pricking portion, that's so big, if that wasn't as big it would be a little easier.* |
|  |  | TASSO-20 – Timepoint #2 | *I'm guessing the size of it. I don't know. When I say the size of it, the top piece is a bit bulky. I don't find it's any convenience, but if you're talking about design features, making it slightly smaller.* |
|  | Stronger adhesive | TASSO-7 – Timepoint #3 | *I would say maybe the adhesive needs to be a little stronger* |
|  |  | TASSO-5 – Timepoint #2 | *Just some way to make the adhesive stickier, or an offering to a piece of tape if it feels like it's going to be loose.* |
|  |  | TASSO-5 – Timepoint #3 | *Put more glue on it. It fell off… Put more stickiness on the back of that thing. That's all, just more stickiness. That's all I need. That's the only way to make that device better. More stickiness.* |
|  | Allow users to write date and time directly on device | TASSO-4 – Timepoint #3 | *Very, very comfortable using it. The difficulty comes in having to peel the labels and place the labels, and that's cool too, but for me I'm like, "Why isn't the labels not just built on the damn thing so I just have to put the paper... I mean, just write directly on it?"* |
|  | Give people choice of device color | TASSO-11 – Timepoint #1 | *I don't like the color red on the device. I think that should be a total different color. Red means excitement, it means blood. And you don't see any blood, especially after using it and seeing the video. All you see is a dot. So I think it can cause a little anxiety. Any other color. The color red or anything that has to do with red, I think it should be done away with in my personal opinion.* |
|  |  | TASSO-2 – Timepoint #2 | *Because red is a real... Like, "Stop," and it scares you. And blood is the color of red and a lot of people faint. Some people, 'I can't be a doctor, if I can't look at blood." Do you know what I mean? … They give casts now, if a person breaks their arm, they ask them what color tape they want the cast.* |
| Device kit | Larger alcohol pads | TASSO-11 – Timepoint #1 | *I don't like the alcohol squares that you guys use. I think they're too small. Even though you gave us two in the kit, there's some that are bigger, that are more wetter because you're putting them side by side one another to let it draw, but why not have a larger wipe? So you can wipe that area because the wipes, I checked the date on them. They weren't outdated wipes, but they weren't very dry. I mean, they were very dry. There was some moisture there. Yes, but you need it both, but I didn't feel comfortable. So I went and got one of mine.* |
|  |  | TASSO-11 – Timepoint #2 | *I have been given larger alcohol pads. You guys give the real small ones. Whatever kind that I have been given, the new one, the larger pad, those are super duper moist. So it cleans the area really good.* |
|  | Higher quality band-aid | TASSO-20 – Timepoint #2 | *I replaced the band-aids with my own.* |
|  | Include viral load journal or diary | TASSO-4 – Timepoint #3 | *So, that's a good idea to be able to have a home viral load journaling type kit.* |
|  | Make device kit easier to open | TASSO-8 – Timepoint #1 | *I remembered that. I had to use some knife or a scissors to open the first package of that, not the first one, the plastic with it looks like aluminum. It's kind of hard at paper to punch it and rip it. So I think if you guys make some little punch in the side, I don't know what it's called, but something like that, it would make it easier to open it, all the package.* |
|  | Non-stick foil to return device | TASSO-9 – Timepoint #3 | *I don't know if there's some way you could use a non-stick inside when you go to put the used devices back in. Sometimes it's a pain to get them in that bag. Once the tab is removed from the back after you use it, because they stick to the inside of the bag. So I don't know if there's some way you can use like something to prevent that from happening.* |
|  | Include warning that device is not a toy | TASSO-11 – Timepoint #2 | *I think the actual chamber that's down the bottom, that could be a different color and a warning that this is not a toy, on there… Because like I said it does look like a toy, keep out of the reach of children. This is not a toy… Medical device, not a toy.* |
|  | Include Tasso contact in case of questions | TASSO-10 – Timepoint #3 | *I think maybe on the Tasso part that they put contact information there, so if someone has a question or a concern, that they can contact the Tasso people.* |
| Other suggestions | Provide back-up in case of malfunction | TASSO-4 – Timepoint #3 | *So, instead of just two you might have to send three out, because one of three are not going to work.* |
| **Suggestions to Improve the Device Research Experience** | | | |
| No suggestion | | TASSO-10 – Timepoint #2 | *I can't see where there would be any room for improvement there.* |
|  |  | TASSO-11 – Timepoint #2 | *Stay the same as they are.* |
|  |  | TASSO-1 – Timepoint #2 | *No. I mean, right now it is as easy as it can be. It's just like A-B-C.* |
|  |  | TASSO-1 – Timepoint #3 | *No. I think they did everything they could do.* |
|  |  | TASSO-2 – Timepoint #3 | *I really don't think... it is so simplistic that, personally, I don't see anything else that could have been done.* |
|  |  | TASSO-3 – Timepoint #2 | *It's very comfortable working with them [research team], very comfortable. I have no complaints about what it is that they're doing, none whatsoever. I love working with them because they make it fun and they make it easy to deal with because any questions that I have, they're able to answer it. If they can't answer it, their direction to get the answers are always on point. I've never had to wait around for anything when it came down to this trial, nothing. Everything was just spot on and on point.* |
|  |  | TASSO-3 – Timepoint #3 | *No, they did everything. They did everything.* |
|  |  | TASSO-4 – Timepoint #2 | *I don't exactly know because they were pretty good. They were thorough in explaining the device and just the fact of the research in the device and they did a good job of showing me how I operate it.* |
|  |  | TASSO-5 – Timepoint #2 | *I guess because we talked so much, I was comfortable every step of the way. So I can't see where they can improve, because to me they did a great job. For me if an appointment had to be rescheduled or canceled, that they would make arrangements for it. They've always accommodated me. They've kept me informed of everything every step of the way. So I can't really say they could do anything better, because what do I want them to do?* |
|  |  | TASSO-6 – Timepoint #3 | *Everyone has been, I believe on top of things during the whole trial… I really believe that the team has, and everyone that I've been encountered as far going to study has really been helpful and engaging and easy to deal with. So I don't see any negatives in that.* |
|  |  | TASSO-15 – Timepoint #2 | *Nothing really. It's pretty accurate. It's self-explanatory.* |
|  |  | TASSO-15 – Timepoint #3 |  |
| Provide more devices for practice or additional back-up devices | | TASSO-18 – Timepoint #2 | *The more you do it, the better you get at it. If there was maybe some, I know that's probably not cost-effective for you all, but if you have some practice devices, so people could practice and then get better at it… Just some throwaway practice devices.* |
|  |  | TASSO-6 – Timepoint #3 | *Or, maybe they could give you an extra one or two.* |
| Text reminders for when to use device | | TASSO-20 – Timepoint #2 | *Outside of like to make things a little easier, maybe. So every time I have an in-office appointment, I get a text reminder saying, "Hey, your appointment's coming up, meet here at this time." If there was some type of automatic reminder, to say your date to do the TASSO device is coming up.* |
| Mail device kit at home | | TASSO-14 – Timepoint #2 | *Again, I really can't think of anything else. It was like self-explanatory once I watched the video and read the pamphlet. The average person should be able to do this with no issue whatsoever. I can't think of nothing to change it. Other than you want to deliver to my house and I just do the package that way, because I have to go to the clinic. That's the only thing I can see to change, but other than that, no.* |
| Provide a map of nearby private couriers | | TASSO-20 – Timepoint #2 | *So I think something I've done myself, but to have some type of geographical map available of FedEx drop boxes or alerts to something to locate for other participants.* |
| **Experiences with Mailing Blood Samples** | | | |
| Positive experiences | Same-day shipment – plan to go out on day of blood draw | TASSO-11 – Timepoint #2 | *I don't do mine unless I know I'm going out.* |
|  | Availability of private couriers | TASSO-10 – Timepoint #1 | *There's a FedEx just a couple blocks from my house so it worked out perfect.* |
|  |  | TASSO-10 – Timepoint #3 | *I live in the city, so there's a FedEx box on every other corner.* |
|  |  | TASSO-11 – Timepoint #2 | *Only because I do live near a FedEx facility. But I still have to go. It's not like I got to go far but that's six blocks I wouldn't have to go.* |
|  |  | TASSO-1 – Timepoint #2 | *Actually, it's maybe about three blocks from here, from where I live at.* |
|  |  | TASSO-2 – Timepoint #2 | *The box in my neighborhood is about two miles.* |
|  |  | TASSO-18 – Timepoint #3 | *My post office is maybe four blocks from me, so I live close to the downtown district of my area. I don't have far to go to find the drop boxes, but maybe for other people who would have to go a longer distance, that might be an issue for them. Fortunately, I live in an area where it's close by.* |
|  |  | TASSO-3 – Timepoint #1 | *I know right where I live at there's a FedEx box not far from me, so I just took it to the box, dropped it in and walked away.* |
|  |  | TASSO-4 – Timepoint #3 | *I'm actually lucky. I have an actual FedEx store and a Walgreen's that has a FedEx drop off point close to the house. So, that wasn't a problem.* |
|  |  | TASSO-5 – Timepoint #3 | *However, with teleworking, I'm now a mile away from the closest FedEx drop box.* |
|  |  | TASSO-20 – Timepoint #1 | *There are so many FedEx drop boxes in my neighborhood.* |
|  | Pre-labeled or pre-paid | TASSO-1 – Timepoint #1 | *It’s an easy process. I think y'all made it real easy. All just once I do my part, I put it in the app. Put everything in the box and put it in the envelope and I just go drop it off. At a FedEx drop off. And lucky for me, I have one right around the corner from me. So that makes it real easy.* |
|  |  | TASSO-1 – Timepoint #2 | *Everything is right there. I mean, all I got to do is do my part, put it in the bag, seal it up and drop it off. That's pretty easy for me because I don't have to fill anything out. Once I drop it in the bag I know all I got to do is go drop it off and I'm happy with that. Can't get no easier than that.* |
|  |  | TASSO-3 – Timepoint #2 | *It's all helpful because they give you everything. I don't have to look for anything. Everything is already set and ready and prepped, the bags, all the boxes and labels, everything is all there. All I have to do is do the device, put it in the bag, seal it, and get it to the place. So it's easy getting it because where I'm at in Philadelphia it's a lot of FedEx drop boxes. So I have no issues with trying to find a place to drop it at.* |
|  |  | TASSO-4 – Timepoint #3 | *From the whole way it's packaged, that's amazing. It comes in two boxes, it's already got the package, the bag to go in, that is good.* |
|  |  | TASSO-5 – Timepoint #3 | *The fact that the package is already done, like the envelope, the label tag. It's already done. It's pre-done. I don't have to fill that thing out. Perfect. That's it. I'm good.* |
|  |  | TASSO-20 – Timepoint #2 | *Everything is pre-labeled, and it comes with its own shipping package so that makes it extremely easy. I mean, all they have to do is do the device, pack it up and take it to the box, which all of that is simple enough.* |
| Difficulties or concerns | Missed evening drop off time | TASSO-6 – Timepoint #1 | *I had missed the one run that makes it express but she said it would be picked up first thing in the morning. She didn't give me any hassle. All she had to do was sit there and point her little gun at the thing and take a picture, and that was the end of it.* |
|  | No weekend pick-up | TASSO-8 – Timepoint #3 | *Like if there's some times I drop it off at FedEx on Saturdays, but at the new locations specifically, they just pick it up Monday morning.* |
|  |  | TASSO-12 – Timepoint #3 | *Well, the closest one to me is less than a mile, it's certainly not the distance. One thing I did notice is, there doesn't seem to be any weekend pickup. Because one time I dropped it in there on a Friday and I took the time to read that schedule. I had already dropped the package, but it says six o'clock, Monday through Friday. But towards no pickup on Saturday, no pickup on Sunday.* |
| **Communicating Viral Load Test Results and Desired Sensitivity** | | | |
| Communicating test results | Patient/participant web portal or app | TASSO-10 – Timepoint #3 | *They have all these portals now. I would say just through a portal, because that's how I get them now. I get on the hospital and I sign in, and I can look at any of my results. So, I would say attach it to something like that.* |
|  |  | TASSO-2 – Timepoint #3 | *We get a notification about new labs or new results… I think the computer spits that out instantly, and it automatically uploads. I think we're as real-time as we can get… In the computer, in the system. In myPenn, that’s Tasso versus Direct Collection. That’s what they call that, when you go to the lab and get it.* |
|  |  | TASSO-9 – Timepoint #3 | *I mean, if they have access to the app that I'm using through the University of Penn, that would be fine. It's hard to reach me by phone cause of my schedule and I can't answer the phone if I'm logged into work.* |
|  |  | TASSO-18 – Timepoint #2 | *So you have this online account, and once you set it up, it confirms your appointments. Because of COVID, they had all those prescreening questions, and it also lets you know of upcoming appointments and whatever screenings and results.* |
|  |  | TASSO-18 – Timepoint #3 | *I like the portal. Yes. An email that tells me I have the results and then I have to log in to that website. It's the Penn Med Portal. Some people like text messages, but I don't really like everything on my phone. I don't even do a lot of emails on my phone.* |
|  |  | TASSO-3 – Timepoint #3 | *I had the web portal, which is connected to my doctor or to the facility... And then it comes straight to me and via email. So yeah, the communication is good.* |
|  |  | TASSO-4 – Timepoint #3 | *The portal apps. There should be an app that says yes we have your results and this is your numbers. That would make it so much more convenient instead of having to wait for your clinic to tell you. Or your clinic could post them for you on the portal that they have set up…*  *Practically everyone has got a smartphone. So it's easy and very private. What's this app do? Oh, that's my medical records, you don't need to touch that app. You know what I mean?* |
|  |  | TASSO-6 – Timepoint #3 | *Well, it's nice to be able to go into a little Patient Portal and all of that kind of stuff, but I myself, I think that kind of information should be given through your doctor.* |
|  | Email | TASSO-11 – Timepoint #3 | *Personally, I prefer an email, but I think that I know my doctor's office. I know he's going to call me anyway, but he's still also, the office always sends an email, and I also get a phone call. My doctor's office is very big on communication. He wants to make sure that I know what's going on.* |
|  |  | TASSO-18 – Timepoint #3 | *I guess an email, but then an email with attachments that keep things private.* |
|  |  | TASSO-20 – Timepoint #3 | *I would probably say email because I would hate for someone to see. I'm really open with my status and I don't mind, but if I wasn't as open, I wait for like a text message to pop up and someone could see over my shoulder and they're getting information, or a letter. Something like that. Email, and I say email because there's a record of it versus someone to tell me and I have no record of it, so I would say that would be the best way for me.* |
|  | Phone | TASSO-1 – Timepoint #3 | *I prefer phone. I like to talk to people.* |
|  | No text message | TASSO-18 – Timepoint #3 | *Definitely no text message.* |
|  | Doctor’s involvement | TASSO-7 – Timepoint #3 | *And if I have to see my doctor and they give me a review, then I've got to see my doctor too.* |
|  |  | TASSO-3 – Timepoint #3 | *You can have it personally sent to you or yeah, you can always go to the doctor and let them explain to you what was going on. And that's the good thing. I can ask questions about what I'm seeing and get the information that I need from my doctors.* |
|  |  | TASSO-6 – Timepoint #3 | *I think that the doctors should be the ones that at least discuss it with you. So that if you have a question, right then and there, there's someone to answer it.* |
|  | Comparison of viral load from lab versus device | TASSO-1 – Timepoint #3 | *I'd like to be able to see the difference in what is what. How close is this device to what they actually taken in the lab.* |
|  |  | TASSO-2 – Timepoint #3 | *That’s an in-person collection. And then a Tasso would automatically say device, self kit, self-test kit. I think something like that would be in the computer versus that.* |
| Desired sensitivity | As sensitive as possible | TASSO-11 – Timepoint #3 | *I like it being super sensitive. For me, yes, because if it's super sensitive, it just means to me that I may need to do something a little different. My Doctor may need to adjust my medicine in some sort of way, even if it means increasing it or switching brands, or just maybe switching times and I might need to take it a little closer than what I normally do. I think it's very important.* |
|  |  | TASSO-20 – Timepoint #3 | *I would want it to be as sensitive as what I would do in a clinic. If it's going to replace the going to the clinic I need to have as accurate of a reading as possible.* |
| **Devise Use Beyond ATIs** | | | |
| **Perceived Importance of the Device and Testing Viral Load from Home** | | | |
| Would use on regular basis | | TASSO-10 – Timepoint #3 | *It's important to have, obviously, regular tests because you want to see if there's any fluctuation or anything. Obviously, for me, the more the better, the more information I have.* |
|  |  | TASSO-4 – Timepoint #3 | *A resounding hell yeah!* |
|  |  | TASSO-6 – Timepoint #3 | *But if I had to, yes, I could see myself doing it every day, if it meant keeping me alive. And that was my dose of medicine or whatever have you, versus taking a pill... If I had to I would.* |
| Would use on regular basis | No need to take time off work | TASSO-10 – Timepoint #3 | *It would save time and energy. You can literally... You wouldn't even have to take off work to do it. If you got to go to the doctor, sometimes you got to take a half a day off of work.* |
|  | No need to go to doctor’s office | TASSO-1 – Timepoint #3 | *That way, like I said, don't have to go into the office. And it's quick and easy. Sometimes, you may not feel like going in the office, and you can just do it yourself and then send it off. Just got to wait for the results.* |
|  | No need to pay for parking | TASSO-9 – Timepoint #3 | *Less parking I have to pay for every time I have to go down to the city. It's not cheap down there.* |
|  | Monthly | TASSO-4 – Timepoint #3 | *The stick fatigue would set in… Once a month would be great. But we've been doing it weekly, biweekly, that's a bit much doggone it.* |
|  | Would need to be covered by insurance | TASSO-2 – Timepoint #3 | *Hypothetically speaking, if it's covered by insurance and it's ordered like... you have to have an order for things, right? Like a prescription.* |
| Testing viral load from home | Additional source of health information | TASSO-4 – Timepoint #3 | *Like I said, if my doctor says, "Hey, I need to check your viral load a little bit more close because you had a bounce back, you had a resurgence in viral load. Last month we had you at four and this month for some reason you're at 50, because your viral load is going to change throughout time. But 50 is a pretty big jump so we want to see what's going on, if you're going to rise faster maybe it's something you're doing that's counteracting your meds."* |
|  |  | TASSO-15 – Timepoint #3 | *You need to keep a track for your own personal use and for you to know what's going on with your body.* |
|  | Opportunity to ask questions about viral load | TASSO-3 – Timepoint #3 | *Like I said, [the device] is a help to be able to see how the virus is working in your body. Learning more about it and what all of that means as far as the viral load and all of that. Because I learned a lot in these last couple of weeks about that whole viral load due to the spike in the number a little bit. So, it gave me the opportunity to ask questions because being undetectable, I didn't ask questions….* |
|  | Would still require assistance to interpret results | TASSO-5 – Timepoint #3 | *No, only because I believe that if you're testing or if you're looking at numbers, you're not a scientist. You haven't been trained in how to interpret the data. Don't go out there being Dr. [Name] because you're not Dr. [Name]. Dr. [Name] of person went to school for this. [Name], the nurse, went to school for this. This is important. Let's see what the professionals say.* |
| **Possible Effects on Stigma and Social Risks** | | | |
| Positive effects on stigma | Normalizes HIV | TASSO-8 – Timepoint #2 | *I think so because people would maybe think that it's not that complicated to treat the HIV. It is not something hard and that there is technology to help deal with nowadays. So it probably would help [people’s perceptions of HIV].* |
|  |  | TASSO-10 – Timepoint #2 | *It's been normalized, which is good… With this it would be the same. People are just like, "Oh okay, he's getting his blood work done." That's a good thing… Blood work is just a fact of life for everybody. No matter what their issue is, people get blood work. A home system would just be like, "Oh, okay. That's how we do it now."* |
|  | Reduces doctor’s visits | TASSO-1 – Timepoint #3 | *Because you're not actually going into an office, so you don't have that fear or that worry that somebody's going to see you. You just do everything from the safety of your own home. Nobody knows what is what.* |
|  |  | TASSO-2 – Timepoint #2 | *Yeah, I want to qualify my yes. The reason is, a lot of the people that you go in laboratories with, even though there's HIPAA laws, people talk. And if you go somewhere and you have tubes to be drawn, every single one of the phlebotomist know what those tubes are because they're taught. And if you just happen to be getting a blood draw from a phlebotomist that knows your family, or knows friends of your friends, y'all have people in common, that becomes a threat to your security as a person and your privacy.* |
|  | Helps with disclosure of undetectable HIV status | TASSO-18 – Timepoint #3 | *I was uncomfortable with the U=U meaning undetectable, untransmittable. When that first was introduced, because I thought that could send the wrong message to people who aren't practicing safe sex. If I was able to test at home and get my viral load at home and show that to a partner and educate them, jeez, do you know how easy that would be to reduce the stigma and the fear that people have that they can't engage in romance and stuff like that. It really helps with your disclosure to people who you are being intimate with.* |
| No effect on stigma | Private device | TASSO-10 – Timepoint #2 | *No. It's usually done in the privacy of your home, not that I'm doing it around people.* |
|  |  | TASSO-15 – Timepoint #2 | *I don't think so. I'm thinking it would just be the same. I don't see what the difference would make. You're just be in the privacy of your home doing it. Now that would be like a private person instead of going to a clinic.* |
|  | Stigma still pervasive | TASSO-3 – Timepoint #3 | *So, what people don't know, they'll never be able to understand if they don't learn about it. So I believe there would still be some sort of stigma about it because of ignorance. People just don't know.* |
|  |  | TASSO-12 – Timepoint #3 | *Well, just people have their opinions.* |
| Possible social risks | Precautions with device (e.g., bag) | TASSO-18 – Timepoint #2 | *If people are seeing me leave my apartment with that thing under my arm on a regular basis, then that would draw a little attention that I wouldn't want. But keep in mind that I'm super, super discreet and private and paranoid about it. So in the future, I probably won't carry my FedEx bag under my arm, physically. I'll probably throw it in a grocery bag so no one sees what it is. Even though that's super paranoid and probably unnecessary, that's just me.* |
| Confidentiality and private | No HIV identifier | TASSO-8 – Timepoint #2 | *Because doesn't have any kind of information saying that on the package or anywhere else.* |
|  |  | TASSO-10 – Timepoint #1 | *There's nothing in there that says anything.* |
|  |  | TASSO-11 – Timepoint #2 | *No, that was brilliant because it doesn't say blood sample on side the envelope at all. Nobody knows what's in the box.* |
|  |  | TASSO-3 – Timepoint #2 | *Because it has nothing that I think that has my name on it. None of that. It's just a number. It's just a number to the device. The number is connected to me and only ones that know that number and connection to me is you guys, my nurses and stuff like that.* |
|  |  | TASSO-4 – Timepoint #1 | *Not labeled. It only says sample. It doesn't say why. So, if you have the kit and you don't want anybody to know, you'd be like, oh, that's just some medical stuff I got.* |
|  |  | TASSO-5 – Timepoint #1 | *No, because it's inside the FedEx box. So even if I have company come over, there's nothing to identify any personal issue in my life in my home… I don't want to advertise my status, but that's not something that would give it away.* |
|  |  | TASSO-5 – Timepoint #2 | *And remember, it's only collecting blood. They don't know how they're collecting blood for diabetes nowadays, which is so common. "Hey, what you doing over there, love?" "Oh, checking my blood level." "Oh, okay." Keep it moving.* |
|  |  | TASSO-6 – Timepoint #1 | *No, because myself looking at it, I don't see anything that says HIV and I don't literally mean just the label, I mean, looking at the device itself. It's a collection device. The person could have diabetes. The person could have hemophilia. The person could have so different things, so I don't think that that's an instant cue to people.* |
|  |  | TASSO-6 – Timepoint #3 | *I want them to, but I don't really think that this device in any way is going to out anyone for HIV. That's my personal opinion. I'm certainly not worried about it, not for me, because most of the people around me are more educated than that.* |
|  |  | TASSO-20 – Timepoint #2 | *I wouldn't be concerned about it because you just see this box. If you go into the box, you see the device but it doesn't spell out that this is for HIV. If someone wants to do the research and look into what it is, then that could become a concern. But otherwise, no. Especially because we know that people draw their own blood for different reasons. So it's not that big of a concern.* |
|  |  | TASSO-12 – Timepoint #2 | *There's no identifying indicators on the device or the packaging.* |
|  |  | TASSO-15 – Timepoint #2 | *Anybody could use it with any kind of sickness, gay, straight, whatever the case may be, whatever health issues.* |
| **Additional Potential Uses for the Device** | | | |
| Additional tests | CD4 or CD8 | TASSO-8 – Timepoint #3 | *Take it for CD8.* |
|  |  | TASSO-20 – Timepoint #3 | *As it relates to HIV, my CD4 as well as a viral load.* |
|  | Chemistries | TASSO-5 – Timepoint #3 | *The CBC and all that stuff.* |
| Additional conditions | Blood sugar | TASSO-10 – Timepoint #3 | *Testing your sugar, any of those things that can be done in the privacy of their home. And then, they can deal with the results with their loved ones or themselves, whatever, without having to be in a clinical setting and getting information.* |
|  |  | TASSO-5 – Timepoint #3 | *Absolutely. You can collect insulin tests, diabetes. That's the first one I thought about. I mean, of course now they've got that little pen that they stick in their finger, but if you've got to collect some cells for the blood test, I mean the sugar test.* |
|  |  | TASSO-6 – Timepoint #3 | *I think in my head I'm thinking that it could be of good use for blood sugar samples or things like that for diabetics.* |
|  | Any other illness | TASSO-2 – Timepoint #2 | *When we talked about it expanding to other populations, the cancer population. It could be expanded for people with other ailments, so it doesn't specifically stand out or scream, "HIV people." It doesn't do that, so nobody would really know that this control group, people like myself, living with HIV, that had a diagnosis of AIDS, so I always have a diagnosis of AIDS.* |
|  |  | TASSO-3 – Timepoint #3 | *So, I think that the device could be used for people such as that or who needs to check their blood for other issues that may be going on. That blood could be a blood sample can be used for. So it's always other things that it could be used for.* |
|  |  | TASSO-12 – Timepoint #3 | *Well, if they could develop it for other maladies, I think that'd be beneficial for everybody.* |
| Additional populations or settings | Individuals who are hard stick or with small veins | TASSO-2 – Timepoint #3 | *Especially those people that they have to send in a specialist team just to draw blood because their veins are really small.* |
|  | Individuals in nursing homes | TASSO-11 – Timepoint #3 | *I think the people in the nursing homes would love that device.* |
|  | Drug screening | TASSO-18 – Timepoint #3 | *I know that people struggle with passing a drug test. Let's say you're applying for work and you're afraid the marijuana in your system is going to show up in your drug test. I know people who won't fill out the job application or who won't follow through with the second interview because they know that they're not going to pass the drug screening. If this could help people feel safe that there's no longer a marijuana or whatever in their system that can help them potentially get employed.* |
| **Additional Considerations** | | | |
| Hope and optimism | | TASSO-6 – Timepoint #1 | *I hope that this study is a success because I think the devices would be very helpful.* |
| Engage PWH as peer educators | | TASSO-11 – Timepoint #1 | *I think they should use members who are actually living with HIV, because if you're going to be taking money out of the community, you need to put something back in. So just like I'm doing, you put your money where your mouth and there's different places that have peer educators, that go around teaching people about HIV and how to take care of themselves.* |
